# Animal Models for the Study of Rodent-Borne Hemorrhagic Fever Viruses: Arenaviruses and Hantaviruses

**DOI:** 10.1155/2015/793257

**Published:** 2015-07-21

**Authors:** Joseph W. Golden, Christopher D. Hammerbeck, Eric M. Mucker, Rebecca L. Brocato

**Affiliations:** Department of Molecular Virology, Virology Division, United States Army Medical Research Institute of Infectious Diseases, Fort Detrick, MD 21702, USA

## Abstract

Human pathogenic hantaviruses and arenaviruses are maintained in nature by persistent infection of rodent carrier populations. Several members of these virus groups can cause significant disease in humans that is generically termed viral hemorrhagic fever (HF) and is characterized as a febrile illness with an increased propensity to cause acute inflammation. Human interaction with rodent carrier populations leads to infection. Arenaviruses are also viewed as potential biological weapons threat agents. There is an increased interest in studying these viruses in animal models to gain a deeper understating not only of viral pathogenesis, but also for the evaluation of medical countermeasures (MCM) to mitigate disease threats. In this review, we examine current knowledge regarding animal models employed in the study of these viruses. We include analysis of infection models in natural reservoirs and also discuss the impact of strain heterogeneity on the susceptibility of animals to infection. This information should provide a comprehensive reference for those interested in the study of arenaviruses and hantaviruses not only for MCM development but also in the study of viral pathogenesis and the biology of these viruses in their natural reservoirs.

## 1. Introduction 

Rodentia account for nearly 50% of the mammal population and impact humans in at least two deleterious ways: they destroy vast quantities of food per year and they are carriers of infectious disease contributing directly and indirectly to the spread of ~20 viruses and >40 bacteria and parasites [1]. In Asia alone, 5–17% of the rice crop is eaten by rodents. By some estimates, this amount of food is enough to feed >200 million people. In the spread of infectious disease, rodents play two key roles, indirect and direct. Along with other mammals, rodents can function as short-term carriers that amplify infectious agents which are in turn spread to humans by intermediaries, predominantly arthropod vectors [1]. Such indirect roles in virus spread may include amplification of, for example, Yellow Fever virus and Crimean Congo Hemorrhagic Fever virus, which are spread to humans via mosquitos and ticks, respectively, after the insects feed on infected rodents (among other animals). Rodents can play a direct role in the dissemination of infectious viruses to humans by serving as the sole viral reservoirs (or carriers) of the infectious agents in nature. Within these carrier rodent populations, infectious agents are propagated and maintained in perpetuity by vertical (parent to offspring) and/or horizontal transfer (adult to adult) [2]. From these carrier populations, infectious diseases are spread to humans from direct contact with rodent secreta and excreta. Increases in rodent vector population, often because of increases in food sources due to favorable weather conditions, can lead to demonstrably higher instances of viral disease in human populations [1]. Rodents play critical roles as the major reservoir of two virus groups,* Arenaviridae* and hantaviruses (family* Bunyaviridae*) [2].

*The Importance of Animals Models in the Study of Arenaviruses and Hantaviruses*. Both arenaviruses and hantaviruses are continually emerging and reemerging zoonoses around the world. In addition to naturally occurring infections, arenaviruses are also considered biological weapon threat agents [3]. Several members of these virus groups can cause significant disease in humans that is generically termed viral hemorrhagic fever (HF) and is characterized as a febrile illness with an increased propensity to cause acute inflammation resulting in varying degrees of vascular leakage and shock [4]. Accordingly, there is an increasing interest in understanding the biology of these viruses and also an interest in the development of medical countermeasures (MCM) to mitigate these threats. Methods to control the threat these viruses impose upon humans require a broad understanding of virus biology, including understanding how infection within rodent carrier populations leads to the emergence of human pathogens and why some of these viruses are able to subvert immune defenses and establish catastrophic disease in humans. Development of MCMs requires animal models that faithfully recapitulate at least the salient features of the human disease caused by the infectious virus. Here, we review animal models that have been developed for the study of arenavirus and hantavirus biology. Information regarding arenavirus and hantavirus animal models has been reviewed in varying detail elsewhere [5–7]. We expand upon this knowledge and discuss important recent findings, including in-depth analysis regarding lethal and nonlethal animal models. We also highlight infection studies in natural rodent carriers. In addition, we discuss the importance of routes of infection and the impact that strain heterogeneity has on the study of these viruses in various animal models. This information should provide a comprehensive reference for those interested in the study of arenaviruses and hantaviruses not only for MCM development, but also in the study of viral pathogenesis and the biology of these viruses in their natural reservoirs.

*Arenaviruses*. Arenaviruses are enveloped ambisense single-stranded RNA viruses with two segments, small (S) and large (L), encoding a 10.7 Kbp genome consisting of five proteins (reviewed in [8]). The S segment encodes the nucleoprotein (NP) and the glycoproteins GP1 and GP2. GP1 and GP2 are the receptor binding protein (and target of neutralizing antibodies) and the membrane fusion protein, respectively. The L segment encodes the RNA-dependent RNA polymerase and the Z protein. This family is divided into two general complexes, the old world (OW) and new world (NW), based on initial geographical region of virus isolation and serology. Several of the >20 known species of arenaviruses are able to cause disease in humans. Lymphocytic choriomeningitis virus (LCMV), first isolated by Armstrong and Lillie [9], is the most ubiquitous arenavirus owed to its ability to persistently infect mice (*Mus musculus*) and pet rodents including hamsters and guinea pigs [8]. Human disease caused by LCMV is generally mild, often asymptomatic, and death is a rare exception. It will not be a focus of this review. We do point out that, rare as it is, even LCMV is capable of causing HF disease in humans [10]. However, in this review we focus on arenaviruses that more commonly cause HFs and have a markedly higher propensity for lethal disease in humans. Among these, the most prominent human pathogen is Lassa virus (LASV), an OW arenavirus and causative agent of Lassa Fever (LF). LASV causes upwards of 100–300 K human infections per year, primarily in the Mana river region of West Africa (Liberia, Guinea, and Sierra Leone); however, infections also occur in Nigeria and Mali [11, 12]. Several NW arenaviruses also cause HFs. The NW complex is categorized into clades A, B, and C, but only those of clade B are pathogenic to humans [13]. Junin virus (JUNV) is the most prominent pathogen among this group and is the causative agent of Argentine HF (AHF). Machupo virus (MACV), Guanarito virus (GTOV), and Sabia virus (SABV) also cause human disease in Bolivia, Venezuela, and Brazil, respectively. MACV, GTOV, and SABV are the causative agents of Bolivian HF, Venezuelan HF, and Brazilian HF [8, 14]. Other arenaviruses pathogenic to humans have emerged more recently. These include Chapare virus (CHPV) in Bolivia and Lujo virus (LUJV) in Southern Africa [15, 16]. Additionally, another NW arenavirus, White Water Arroyo virus (WWAV), was implicated in human disease in North America [17].

Originally thought to be spread by arthropod vectors (mites) [18], subsequent studies [19] indicated rodents play a key role for arenaviruses maintenance in nature. Individual arenavirus species establish a persistent infection predominantly, though not always, in a single rodent species in specific geographical locations [20]. LF results from human exposure to the persistently infected rodent species* Mastomys natalensis* [21]. JUNV is spread to humans by exposure to* Calomys musculinus*. MACV is spread to humans by contact with* Calomys callosus* and GTOV is spread by two rodent species:* Sigmodon alstoni* and* Zygodontomys brevicauda* [20]. The host of SABV has yet to be identified. Human infection ensues upon exposure to rodent excreta and secreta (urine and saliva) and is generally transmitted through aerosols, skin abrasions, and probably ingestion. Rodent habitat preferences play key roles in the extent by which human populations are impacted by these viruses. The LASV host,* Mastomys natalensis*, is widely distributed in Sub-Saharan Africa and, accordingly, vast groups of humans in this region are at risk to infection. NW arenaviruses are generally considered diseases of agricultural workers, the exclusion being MACV. This can be explained by the fact that MACV persistently infected* Calomys callosus* associate more closely with human dwellings [22], compared to other NW arenaviruses that infect rodents associated more predominantly with rural locations. One NW arenavirus, Tacaribe virus (TACV), was initially isolated from bats, suggesting that bats may also play a role as carrier populations for arenaviruses [23]. However, recent evidence indicates that bats are not competent arenavirus carriers and are only transiently infected [24].

*Arenavirus Human Disease*. Following exposure to infected rodents, in 7–14 days (but up to 21), humans develop proverbial “flu-like symptoms” with fever (>38°C), malaise, myalgia, muscle pain, and abdominal pain (reviewed in [8, 11, 25]). Initial stages of disease are similar for OW and NW arenaviruses. In addition to being infected by rodents, nosocomial and person-to-person spread have been documented for both OW and NW arenaviruses, including LASV, LUJV, MACV, and JUNV [14, 15, 26]. Subsequently, some patients deteriorate with more pronounced gastrointestinal pain, dizziness, headache, retro-orbital pain, vomiting, diarrhea, or constipation. A remittent fever is a universal symptom of arenavirus infection. In some cases, muscle pain becomes incapacitating [27]. At this stage, symptoms from LASV and NW arenavirus infections begin to differentiate. NW arenavirus symptoms often consist of bleeding disorders beginning with minor mucosal hemorrhagic including petechial rashes and bleeding from gums, vagina, and/or gastrointestinal tract [14]. Hemorrhage is a rare occurrence during LASV infection, predominately associated with severe cases [7, 11]. Thrombocytopenia, neutropenia, and leukopenia are common during NW arenavirus infection [25]. Consistent with lower levels of hemorrhage, thrombocytopenia is only associated with severe LASV infection. Human infections by LUJV were associated with thrombocytopenia and bleeding abnormities including gum bleeding and hemorrhage around injection sites [15]. Nevertheless, similar to LASV, bleeding was not a salient feature of disease. Mild neurological symptoms are common to both NW and OW infection and generally include tremors of arms and tongue. In contrast to OW viruses, NW arenaviruses can cause more significant neuropathology including coma, encephalitis, and convulsions. Indeed, NW arenavirus human disease can be characterized as either hemorrhagic (visceral), neurological, or a mix of the two. In some cases, patients succumb to disease while only presenting with neurological symptoms. Cardiac disturbances for both LASV and JUNV infections are well-described and include ST-segment and T-wave abnormalities. Tissue edema, including pulmonary edema, has been observed for both NW and OW virus infection. Liver pathology occurs during both NW and OW infection; more so with the latter and higher AST/ALT are linked with poor prognosis [11, 25]. Viremia is detectable during both NW and OW infection, and higher viremia during LF is a grave sign [28]. For both NW and OW cause of death is enigmatic and multifactorial, but likely to be a result of multiorgan failure and shock. An important finding in NW infection is the association of high levels of TNF-*α* and type I IFN (IFN-*α*) with poor outcomes [29, 30]. Levels of these cytokines during LASV infection do not appear to correlate with outcome, but more work will be needed to fully understand the nature of the cytokine storm and its impact on host survival [28]. NW arenavirus fatality rates approach 30% in the absence of treatment whereas lethality of LF is much lower at ~1% [31]. However, death rates of those with LF who present to the hospital approach 20%. LUJV fatality rates are quite high (~80%), but only five cases have been reported [15]. Arenavirus infection during pregnancy is particularly dangerous and in the third trimester can result in fatality rates of >90% of mothers [11, 25]. In survivors, infection generally causes loss of the fetus. NW arenavirus disease is nuanced, and some patients present with only neurological symptoms or only hemorrhage with little brain involvement while others present with both. Secondary infection can also contribute to mortality [32]. It is important to note that a wide spectrum of symptomology exists, ranging from subclinical or unapparent infection to life-threatening disease. Seropositive humans who never experienced symptoms of arenavirus infection have been reported in both South America and Africa [21, 33]. Reasons for symptom disparity are unclear, but likely involve innate immune responses among other factors. Why arenaviruses cause catastrophic disease in some but result in a far more subtle infection in others is a fundamental question of arenavirus biology.

Antibody production signals the beginning of viral clearance and the absence of antibodies is associated with death. Production of antibodies during NW arenavirus infections occurs 1-2 weeks after infection, with neutralizing antibodies following behind by a week or so [25]. Neutralizing antibody production during OW arenavirus infection is significantly delayed compared to NW arenaviruses not developing until 1-2 months into convalescence [11]. Survival of OW arenavirus infection is thought to involve T cell adaptive immunity, although the protective role of antibody and T cells during primary and secondary protection is far from understood. Convalescence from arenavirus infection is protracted, lasting months, and can be associated with hair loss. A salient complication in the recovery of LF is unilateral and occasional bilateral deafness (~30%) which resolves in half of patients [11]. Few sequelae exist following recovery from NW arenaviruses. There are currently no FDA-approved vaccines, postexposure prophylactics, or therapeutics to prevent disease incurred by arenavirus. However, a live attenuated vaccine termed Candid#1 is currently being used in Argentina to prevent JUNV infection of agricultural workers who are at high-risk of infection [34]. Additionally, convalescent serum/plasma has been shown to provide protection against JUNV, MACV, and possibly LASV [21, 27, 35]. Other studies suggest that ribavirin has a protective efficacy, particularly against LASV [36–38].

*Hantaviruses*. Hantaviruses (from the family* Bunyaviridae*, genus* Hantavirus*) are negative-strand, single-stranded RNA viruses with three segments, denoted small (S), medium (M), and large (L). The S segment encodes the nucleoprotein (N), the M segment encodes the glycoproteins (G_n_ and G_c_), and the L segment encodes the RNA-dependent RNA polymerase [39]. Some hantaviruses express an additional nonstructural (NSs) protein encoded within an alternate open reading frame (ORF) within the N protein-coding region of the S segment. Expression of this NS protein by Puumala virus [40], as well as two other nonhantavirus members of the* Bunyaviridae* family, Rift Valley fever virus [41], and La Crosse virus [42], suppresses type I interferon and nuclear factor kappa B (NF-*κ*B) activity potentially allowing greater viral replication in the absence of interferon. However, among HPS-causing hantaviruses, there is a poor understanding of the function of this NS protein as ablation of the ORF does not interfere with the ability of the N protein to regulate IFN responses [43].

Hantavirus disease encompasses hemorrhagic fever with renal syndrome (HFRS) and its milder form, nephropathia epidemica (NE), along with hantavirus pulmonary syndrome (HPS) or hantavirus cardiopulmonary syndrome (HCPS) [44]. In both diseases, hantaviruses predominantly infect microvascular endothelial cells and create a vascular leakage-based disease by altering the barrier properties of the endothelium [45]. This nonlytic infection renders the endothelium unable to regulate tissue fluid accumulation in the kidney (predominant with NE and HFRS-causing hantaviruses) and in the lung (predominant with HPS-causing hantaviruses) [46, 47]. Prominent Old World HFRS-causing hantaviruses include Hantaan virus (HTNV), Dobrava virus (DOBV), and Seoul virus (SEOV), that have a case-fatality rate up to 15%, with a high level of morbidity. DOBV and HTNV are associated with severe cases of HFRS, and SEOV associated with moderate disease [48]. Puumala virus (PUUV), the etiological agent of NE, has a case fatality rate of <1% [49]. HTNV is found in China, Russia, and Korea, DOBV is found in the Balkans, and PUUV is found in northern Europe, particularly Belgium, Finland, France, Netherlands, Norway, Sweden, and Russia [46, 50]. SEOV, carried by domestic rats, has a worldwide distribution made possible by international shipping [48]. In fact SEOV, originally coined Tchoupitoulas virus in New Orleans, Louisiana, was detected in brown rats captured in the 1980s [51] and again more recently [52]. Prominent New World HPS-causing hantaviruses include Andes virus (ANDV) and Sin Nombre virus (SNV) that have an increased case-fatality rate of 35% [39]. ANDV is found predominantly in the South American countries of Argentina and Chile, while SNV was the name given to the virus responsible for the Four Corners area outbreak in the United States in 1993 [53]. Each of these hantaviruses have specific rodent reservoirs with Old World hantaviruses carried by infected rodents from the genera* Myodes*,* Rattus*, and* Apodemus*; New World hantaviruses are carried by infected Sigmodontinae rodents [54]. A common misconception is that hantaviruses are carried only by rodent species when, in fact, hantaviruses persistently infect many small mammals, not exclusively rodents. In addition to the above named hantaviruses carried by mice, rats, and voles (order Rodentia), hantaviruses have also been isolated from shrews [Thottapalayam virus (TPMV); Asian house shrew (*Suncus murinus*); order Soricomorpha [55]], moles [Nova virus (NVAV); European mole (*Talpa europaea*); order Soricomorpha [56]], and bats [Magboi virus (MGBV); slit-faced bat (*Nycteris hispida*); order Chiroptera [57]]. However, one important distinction that can be made among these hantaviruses is that only those isolated from rodent species are known to be associated with human disease. It is presumed that most hantavirus-related human disease occurs when persons inhale aerosolized excreta or secreta from infected rodents [58, 59] or, in the case of ANDV, by direct person-to-person contact with infected individuals [60]. Furthermore, although rare, documented transmission of virus through an animal bite has been reported [61]. There have been no documented cases of human hantavirus disease following ingestion of hantaviruses, but animal model data suggests that this remains a possible route of transmission [62]. There are currently no FDA-approved vaccines, postexposure prophylactics, or therapeutics to prevent or treat HFRS or HPS [63].

*Hantavirus Human Disease*. Upon exposure to NE or HFRS-causing hantaviruses, there is an incubation period ranging from 2 to 3 weeks prior to the febrile phase that is characterized by nonspecific flu-like symptoms of fever (>38°C), headache, myalgia, malaise, and abdominal pain [48]. Severe cases of HFRS disease are composed of five stages: febrile, hypotensive, oliguric, diuretic, and convalescent. Hemorrhage may occur and presents as conjunctival injection or bleeding of the mucosal membranes. A petechial rash may occur on that palate and axillary skin folds [53]. A characteristic feature of HFRS is albuminuria in the febrile phase. Following this, the hypotensive phase is characterized by thrombocytopenia, vascular leakage, and shock [64]. In the oliguric phase, patients suffering from severe disease will exhibit hypertension, pulmonary edema, and renal failure. Subsequent to the oliguric phase is the diuretic phase that may last months prior to convalescence. In cases of NE, hemorrhagic manifestations are exhibited in a third of cases, with approximately 5% of patients exhibiting gastrointestinal bleeding or disseminated intravascular coagulation [49]. During severe HFRS, almost half of the deaths that occur happen during the oliguric phase, while a third of deaths occur during the hypotensive phase [53].

Human HPS cases share many of the same clinical signs as HFRS but vascular leakage is focused on the lung rather than the kidneys. Incubation times known for the two most common HPS-associated hantaviruses, ANDV and SNV, can vary between 9 and 33 days but average just under three weeks. Subsequently, the phases of human HPS progress fairly rapidly, often lasting days rather than weeks. During the 1-2 day febrile phase, tachypnea and tachycardia are common, and symptoms may include gastrointestinal signs or severe abdominal pain [65] while laboratory findings indicate leukocytosis, thrombocytopenia, elevated hematocrit, circulating immunoblasts, abnormal liver function, and proteinuria [48, 64, 65]. Disease culminates in the cardiopulmonary phase characterized by cough, dyspnea, tachypnea, hypotension, and pulmonary edema which can result in cardiogenic shock and death within hours. In mild HPS cases, supplemental oxygen may be sufficient to treat patients but, in more severe cases, extracorporeal membrane oxygenation (ECMO) is necessary. Renal failure also occurs in approximately half of HPS patients [64].

The mechanism of HPS pathogenesis is poorly defined but much has been made of correlations between the kinetics of disease and the immune response to hantavirus infection. Increases in neutralizing antibody titers often occur concurrent with the onset of cardiopulmonary phase and, in cases of human ANDV [66] and SNV [67] infection, appear to correlate favorably with disease outcome. However, in severe cases, IgM and IgG responses appear insufficient to prevent viremia or occur too late to prevent disease. Moreover, tissues collected at autopsy of patients succumbing to HPS show localization of increased numbers of T cells and, in general, cytokine-producing cells in the lung at a higher level than that found in the kidney or liver. This finding has implicated cytokine production as a contributing factor to HPS pathogenesis [47, 68, 69]. Furthermore, during the acute phase of HFRS and in fatal HPS cases, cellular infiltrates consisting of disproportionately large numbers of activated CD8^+^ T cells have been reported and genetic correlations between disease severity and HLA haplotype have been observed in patients with milder forms of HFRS and HPS [70–73] leading many to propose mechanisms of disease focused on T cell mediated immunopathology [73–77]. Aberrant levels of vascular endothelial growth factor (VEGF) may also contribute to pathology. Gavrilovskaya et al. demonstrated increased levels of VEGF in the pleural edema fluid (PEF) of HPS patients. VEGF is known to be a potent regulator of vascular remodeling via the coordinated disassembly and reassembly of adherens junctions. Notably, the observed increase in VEGF in PEF as well as PBMCs correlated with HPS disease severity, with the highest levels observed in a fatal HPS case [78]. The role of cytokines and immune cells in the severity of hantavirus disease is a major area of investigation.

## 2. Arenavirus Animal Models

### 2.1. Guinea Pigs

Guinea pigs (*Cavia porcellus*) have been a model for arenavirus infection studies since at least 1965 [79]. These rodents are highly susceptible to infection by both NW and OW arenaviruses, with LD50 values for some strains of JUNV and LASV as low as 1-2 PFU. Two strains of guinea pigs have been employed, inbred strain 13 and outbred strain Hartley (reviewed [6]). Disease can be produced in guinea pigs challenged by multiple routes including intraperitoneally (i.p.), intranasally (i.n.), subcutaneously (s.c.), intracranially (i.c.), intramuscularly (i.m.), aerosol, and oral routes [80–82]. Among these, s.c. infection with LASV and i.p. infection with NW arenaviruses are the predominate routes of infection chosen by investigators. Important differences in strain susceptibility and disease course exist for NW and OW infection of guinea pigs.  Table 1 lists commonly used strains of arenaviruses and their providence. Prominent arenaviruses animal models are listed in Table 2.

In general there are no significant differences in NW arenavirus disease courses between the strain 13 and Hartley guinea pigs and viruses lethal in one strain are equally lethal in the other [83]. Most guinea pig data has been generated using JUNV. In humans, disease course can be hemorrhagic (also called “common” or “visceral”), neurological, or a mixture of the two and these variances can be produced in infected guinea pigs [81, 84]. Disease manifestation is extremely dependent on virus strain and some strains, such as Romero and XJ, cause hemorrhagic disease in humans and result in >90% animal death whereas other strains cause limited animal death or in the case of attenuated strains, such as Candid#1, no death [6, 81, 82, 85]. Other JUNV strains, such as P3827, produce a predominantly neurological pathology with no hemorrhagic manifestations. Hemorrhagic disease is characterized by weight loss and fever, culminating in shock and death [81, 86, 87]. Several disease signs mimic human disease including development of thrombocytopenia, neutropenia, and leukopenia, in addition to elevated AST levels [83]. Hemorrhagic-causing strains of JUNV cause viremia and virus replication occurs in the spleen, lymph nodes, and bone marrow. Gastric hemorrhage and bone marrow necrosis were also common features. Neurological disease causing strains generally results in encephalitis with hind-limb paralysis being a common finding [81]. Virus can be found in the brain following infection with both hemorrhagic and neurological causing viruses, but only infection with the latter typically results in moderate polioencephalitis. Death from hemorrhagic causing strains generally starts in the second week, with death from neurotropic strains being delayed until the third week. Similar to humans, infected guinea pigs produce type I IFNs, especially IFN-*α*2 which continually increase as infection progresses and may be involved in pathology [88]. As a further demonstration of congruency between guinea pig and human arenavirus disease, about 10% of JUNV-infected humans treated with anti-viral antibody develop a late stage neurological disorder and this can be faithfully reproduced in the guinea pig model [80]. While disease in guinea pigs is similar to humans, some differences exist, in particular disease is much more aggressive in guinea pigs, and, for example, bone marrow necrosis while less common in humans is very common in JUNV infected animals. Dissimilar to nonhuman primates (NHPs), JUNV strains causing neurological or hemorrhagic human diseases do not necessarily cause the same syndrome in guinea pigs; in general, infection in guinea pigs is skewed towards hemorrhagic disease.

The JUNV/Hartley model system has been used to evaluate antibody-mediated protection [80], vaccines [89–91], small-molecule inhibitors [92, 93], and pathogenesis [81, 83]. JUNV strain Romero has emerged as the preferred strain for infection studies. This includes more recent studies showing subunit virus vectors vaccine consisting of Venezuelan encephalitis virus encoding the JUNV glycoprotein precursor gene protect 100% of guinea pigs from lethal strain Romero challenge after two vaccinations [90], in addition to studies demonstrating the protective efficacy of the RNA-dependent RNA polymerase inhibitor Favipiravir T-705 [92]. We have also shown that potently neutralizing antibodies produced by DNA vaccination in rabbits can passively protect guinea pigs (100% survival) when administered one day prior to or two days after challenge (Golden, J. W., and Hooper J. W., manuscript forthcoming).

Other NW arenaviruses cause a disease in guinea pigs similar to that induced by JUNV. To date, NW arenaviruses causing lethal infection in guinea pigs are JUNV, MACV, and GTOV (Table 1). Wild-type MACV lethality ranges from 20 to 80% (strain Carvallo) [6, 94–96]. Mortality of MACV passaged five times through guinea pigs approaches 100% [6]. Recent findings indicate that MACV strain Chicava can cause 100% lethality in guinea pigs [97]. GTOV infection of either strain 13 or Hartley guinea pigs is 100% lethal [98, 99]. In our laboratory, SABV failed to produce a lethal disease in Hartley guinea pigs (Golden, J. W., unpublished data). To our knowledge, the lethality of CHPV in guinea pigs has not been tested. Despite the LD50 being <2 PFU [80], higher doses of virus (>1,000 PFU) are more commonly used. It is not clear why such high doses are typically employed. Lower doses of virus maintain similar disease kinetics and mean time to death (Golden, J. W., unpublished observations), making it unlikely that lower doses would be detrimental to the model. On the contrary, lower doses may prevent masking important protective effects of MCMs by an overtly high infectious dose.

Infection of guinea pigs by OW species is less efficient and highly dependent on the species of guinea pigs (Tables 1 and 2). Initial attempts by Walker et al. to produce a guinea pig LASV disease model using Hartley guinea pigs were abandoned because, while it produced a disease with >60% mortality, it was thought infection in these animals was not similar enough to disease in humans to justify further exploration [110]. Evidence that inbred animals may be more susceptible to arenavirus infection led Jahrling and Moe to reexamine LASV infection in guinea pigs. This group determined that several strains of LASV cause lethality in inbred strain 13 guinea pigs at doses >2 PFU, but infection in Hartley guinea pigs is markedly less lethal (~30%) even with higher doses [102, 109]. Hartley guinea pigs succumbing to disease generally have higher viremia compared to survivors. It is unclear why mortality in Hartley guinea pigs was markedly higher in the Walker (67%) versus the Jahrling study (30%) [109, 110]. LASV also causes lethal disease in strain 2 guinea pigs [109]. Similar to human disease, infectious virus can be isolated from liver, spleen, lymph nodes, salivary glands, lung, adrenal glands, kidney, pancreas, heart, and brain in strain 13 animals. Contrary to human disease, the liver is not a major target of LASV in the guinea pig [109]. Unlike NHP models, there is an incongruity between humans and guinea pigs such that strains in humans that cause severe disease do not necessarily do so in rodents. Strain Josiah is the prototypical strain used in guinea pig studies, but strain Z-132 has also been used and both are 100% lethal (Tables 1 and 2) [101, 102, 109]. LASV strain Sormoba-R is a recent isolate taken from rodents in Mali and displays reduced lethality (~60%) in strain 13 guinea pigs [101]. Similar to LASV, strain 13 guinea pigs are highly susceptible to LUJV lethal infection [108]. The strain 13 guinea pigs used for LUJV infection studies were aged 52–78 weeks at the time of infection. This contrasts with the younger animals typically used in LASV studies which are <16 weeks old. LUJV infection is characterized by anorexia, fever, and liver pathology. Strain 13 guinea pigs have abated hypersensitivity reactions to some antigens and reported delays in humoral immune responses when compared to Hartley guinea pigs [145]. Whether these factors account for the enhanced lethality of LUJV and LASV infection in these animals will require further inquiry.

Because they reasonably recapitulate the salient features of human disease, guinea pigs have become the preferential small animal model for arenavirus study. Despite the fact that similar disease is produced in strain 13 and Hartley guinea pigs infected by NW arenaviruses, preference is on the latter given that Hartley guinea pigs are more readily available. Additionally, Hartley guinea pigs are outbred and, as such, may provide a more comprehensive understanding of disease progression and countermeasure efficacy in a heterogeneous population such as humans. Because of the increased susceptibility, strain 13 guinea pigs have become the small animal model of choice for OW studies essentially by default. As has been done with Pichinde virus (PICV) (see below), it may be possible to generate LASV and LUJV strains adapted to Hartley guinea pigs. Such mutant strains may shed light on virus factors important to virulence and host susceptibility. It is important to note that the limited susceptibility of Hartley guinea pigs to LASV infection could be exploited for the identification of factors that contribute to LASV lethality.

### 2.2. Mice

Adult mice are susceptible to infection by LCMV and serve as both the natural and experiment animal model for this virus [8]. LASV infection of neonatal mice does not manifest disease; however virus can be isolated from brain, lung, and muscles. Some strains of adult mice infected with LASV by the intracranial route develop an acute neurological disease, while infection by other routes and in other mouse strains lead to no disease [6]. Neither in neonatal nor in adult mice is a disease produced that resembles LF in humans [6]. Adult mice are generally refractory to significant infection by NW arenaviruses [96]. In contrast, infection of neonatal mice following intracranial injection results in 100% mortality, but the disease is unlike that found in humans. Nevertheless, the neonatal model has provided useful information regarding the attenuation of JUNV vaccine strains. Most recently, this model was used to demonstrate that a single point mutation in the transmembrane region of the GP2 glycoprotein is involved in the attenuation of JUNV strain Candid#1, the current vaccine used in Argentina for prevention of AHF [121]. These findings were recently repeated with MACV, and the same mutation leads to viral attenuation in the mouse model [146]. Thus, while not useful for pathogenesis or MCM development, immune intact neonatal mice can provide valuable insight in the factors contributing to the attenuation of NW arenaviruses.

Adult knockout mice have recently been employed as small animal models for LASV, JUNV, and MACV lethal disease (Table 1). MACV strain Carvallo causes a lethal disease in two mouse strains, Interferon *α*/*β*/*γ* receptor double knockout (IFNR KO) mice and STAT-1 single knockout mice [135, 136]. JUNV strain Romero also caused lethal disease in IFNR KO mice [122]. In STAT-1 mice, the route of infection is critical as only the i.p route produced 100% lethality by day 8, the s.c. route resulting in ~60% lethality, and lesser still the i.n. route causing ~30% lethality. STAT-1 mice failed to display extensive thrombocytopenia that is characteristic of NW disease but did display clinical and histopathological similarities, including splenic necrosis. IFNR KO mice succumbed to disease with death from JUNV occurring ~day 14 and MACV ~day 33. Tissue tropism was similar to human disease, but inflammation was markedly more severe. Knockout mice have also been used in the study of LASV disease. Type I IFN *α*/*β* receptor single knockout and Interferon *α*/*β*/*γ* receptor double knockout mice infected with LASV exhibit weight loss, viremia, and elevated liver enzyme levels; however infection in this model is generally not lethal [147, 148]. Similar disease courses were observed by multiple LASV strains. STAT-1 knockout mice have also been shown to be highly susceptible to infection by LASV [149].

Given the importance of IFN and STAT-1 in adaptive immune responses, it is unlikely that these models will be useful for vaccine studies. Furthermore, type I IFN potentially plays a critical role in the pathogenesis of NW arenavirus disease in humans, with higher levels a predictor of lethal outcomes [29]. Lack of intact IFN signaling pathways in both murine models produces conditions inherently dissimilar to that in humans. Overall, these knockout models may be useful for evaluation of immunotherapeutics and small molecule inhibitors of arenavirus infection. Indeed, ribavirin was found to protect STAT-1 KO mice from lethal MACV infection [136]. However, guinea pigs would likely provide the same information at equivalent statistical powers while at the same time producing a more natural disease progression. Despite this, knockout murine models have provided critical insight into factors involved in host susceptibility to arenavirus disease, including the importance of IFN signaling. Another important example of such insight stemmed from use of MHC I knockout mice and mice expressing human MHC I revealed the importance of T cells in the pathogenesis of LF [150]. The availability of reagents for mice and the wide range of transgenic strains will likely lead to more interesting discoveries pertaining to host susceptibility to arenavirus infection.

### 2.3. Hamsters

Similar to mice, infection of neonatal hamsters with human pathogenic NW arenaviruses results in lethal disease [96]. In contrast, immune intact adult hamsters are generally not susceptible. However, a report from the 1960s indicated extended viremia can be supported in hamsters infected with hamster-adapted MACV strains [19]. Indeed, adult hamsters that cannibalized infected neonatal hamsters shed virus in urine and saliva for an extended period of time (~509 days) and in some cases showed neurological signs of disease. It is unclear from this study if inbred or outbred hamsters were used. Inbred hamsters are generally more susceptible to disease and use of these hamsters could explain these observations. Overall, the finding that hamsters can shed virus over long periods of time incriminated rodents as carrier populations that maintain arenaviruses in nature. However, the use of adult hamsters in the study of human pathogenic arenaviruses is limited and generally abandoned.

### 2.4. Persistent Infection Models

Animal models have been developed in the laboratory to investigate the biology of arenavirus infection in rodent carrier hosts. These have included* Mastomys natalensis* (LASV),* Calomys callosus* (MACV), and* Calomys musculinus* (JUNV) ([20] and Table 2). These studies have provided evidence that arenaviruses are maintained in rodent populations by both vertical (parent to neonate/fetus) and horizontal transmission [131–133]. The general consensus is that OW arenaviruses establish lifelong (or at least extremely long-term) infections in fetal and neonatal rodents yet result in only transient infections of adult animals. Infection of fetal animals is lethal for NW arenaviruses, but lifelong infections can be established in neonates [133]. In a seminal study, NW arenavirus infection in adult rodents was studied by infecting* Calomys callosus* with MACV [141]. This study identified two district populations of animals, termed type A and type B. Type A rodents become persistently infected with limited antibody production and virus was isolated from oral swabs, urine, and serum for long periods of time with average titers of 4,800, 8,100, and 37,100 PFU/0.05 mL, respectively. Type B animals developed humoral immune responses, including neutralizing antibodies, and virus became undetectable in the serum. However despite humoral immune responses, type B animals still shed virus through urine and oral swabs for many months, albeit at significantly reduced levels relative to type A animals.

Studies using persistent infection models have also provided some insight as to how mutant viruses infectious to humans may emerge from rodent populations. In* Calomys musculinus* infected with JUNV, mutant viruses appearing in infected host are significantly less susceptible to neutralization by antibody produced against the input virus [134]. Abraham et al. proposed that minor amino acid changes in GP1, the receptor binding protein and target of neutralizing antibody, may allow the virus to interact more efficiently with the human receptor [151]. Together these data may provide a model to at least partially explain the emergence of human pathogens from rodent populations; however, further work would be needed to fully explore this possibility.

As interesting as work in the natural hosts may be, it is counterbalanced with the fact that there is great difficulty in working with these models both logistically and biologically. The need for high-containment animal laboratories and the limited availability of animals which may require capture of uninfected breading pairs [141] and use of their offspring makes these studies daunting. In addition, there are limited reagents available to study the immunological aspects of virus infection in these rodent species. Nevertheless, studies in these persistent infection models could provide important insight not only of carrier state, but also how human pathogens emerge from rodent populations. Outdoor laboratories have been created to study disease caused by Sin Nombre virus (a NW hantavirus) in its natural deer mouse reservoir (see below), demonstrating that such studies are feasible even in high-containment.

### 2.5. NHP Models

Both NW (common marmoset) and OW (macaques) NHP have been used to model NW and OW arenavirus-induced diseases (reviewed in [6]). Arguably, macaques are considered the “gold standard” model(s) of arenaviruses and generally recapitulate human disease. To date, LF, AHF, and BHF disease models in macaques using prototypical strains of LASV (strain Josiah), JUNV (strain Espindola), and MACV (strain Carvallo) viruses have been characterized [106, 111–115, 123–125, 137–140, 152]. These models have played important roles in studies focusing on viral pathogenesis and the evaluation of MCMs including vaccine candidates, immunotherapy, and antiviral(s) [37, 107, 111, 126, 137, 153–159]. Rhesus (*Macaca mulatta*) or cynomolgus macaques (*Macaca fascicularis*) are typically exposed to a viral dose typically ~1,000 PFU via a parenteral route. Macaques have also been infected by the aerosol route with MACV [97], LASV [116, 117], and JUNV [125]. Aerosol exposure generally produced disease indistinguishable from other routes. However, one recent study found that the onset of disease and relative lung involvement were different in cynomolgus macaques infected with MACV (Chicava strain) by the aerosol and i.m. routes [97]. While both LASV and NW arenaviruses cause disease in NHPs, as in the guinea pigs, the resultant disease between the two complexes is divergent.

Rhesus macaques parenterally exposed to LASV develop clinical illness similar to LF, marked by lethargy, anorexia, rash, fever, and death [111, 113]. Unlike cynomolgus macaques, outcome is dose dependent, as lower doses tend to be more lethal (reviewed by [6]). Viremia can be detected by day 5 after exposure and escalates until the animal succumbs to infection. As with humans, higher levels of viremia correlate with more severe disease. Virus can be isolated from visceral tissues, with highest viral loads, contrary to guinea pigs but similar to humans, in the liver. Histopathology of these tissues reveals minor lesions relative to the viral load. Animals are leukopenic, have a slight decrease in platelet counts, and are consistent with liver involvement, raised liver enzymes (ALT and AST). Cynomolgus macaques exposed to LASV (Josiah or Z-132) have a similar clinical presentation, with the exception that facial edema was a prominent early sign and some neurological manifestations were observed [101, 115, 120, 157]. However, not all cases of NHP infection by human pathogenic OW arenaviruses represent a transcript of the human disease. LUJV, lethal in 4 of 5 human cases, produced only mild illness in cynomolgus macaques [120]. Similarly, LASV isolated from a fatal case (strain AV) had an alternative disease course and failed to produce uniformly fatal disease in NHPs [160]. Why some LUJV and some strains of LASV fail to reproduce symptoms in NHPs is unclear.

NW arenavirus infection of NHPs has been extensively examined. NW arenaviruses produce both hemorrhagic and neurologic manifestations in humans. Lending to its credibility, the macaque model has been shown to recapitulate hemorrhagic and neurologic disease when infected with strains causing these variances in humans. Indeed, rhesus macaques exposed to JUNV strains isolated from humans with severe hemorrhagic (Espindola strain), neurologic (Ledesma strain), or nonfatal disease (Romero strain) exhibited manifestations that paralleled the human condition from which they were isolated [6, 123, 124]. Within the first 2 weeks postparenteral injection with the hemorrhage causing JUNV strain Espindola, animals exhibit viremia, weight loss, constipation or diarrhea, edema of the face, flushing, hemorrhage, and a macular/petechial rash [37, 107, 124]. Also, there are decreases in lymphocytes, platelets, and hematocrit similar to that seen in humans. The degree of neurological involvement tends to be strain-specific with most severe symptoms associated with strains that predominately cause neuropathology in humans (Ledesma strain) [106, 123]. Subcutaneous injection of rhesus macaques with MACV (Carvallo strain) results in many of the general clinical phenomena (anorexia, diarrhea, and skin rash), but, for the small percentage of animals that survive the hemorrhagic phase, there is neurological involvement. Although cynomolgus macaques are susceptible to the Carvallo strain of MACV, clinical cues of infection before death were obscure [137]. On the other hand, after intramuscular installation of Chicava strain of MACV, animals developed more signs of human disease, such as rash, fever, depression, hemorrhage, and hematuria [97]. Interestingly, one animal had relatively early signs of neurological disease. Despite attempts to develop this model, no lethal NHP model exists for GTOV [99], and lethality of CHPV and SABV in NHPs have not been explored to our knowledge.

The common marmoset (*Callithrix jacchus*) represents another model host for both OW and NW HFs [118, 127–130]. The small size of the marmoset becomes a great advantage when considering the reduced resources (such as cost, biological containment space, and test article) required for an adequate statistically relevant study. Moreover, the increased experimental need for macaques has hampered the ability of researchers to obtain suitable NHP. For these reasons, both AHF and LF disease have been modeled in the common marmoset. Like the macaque, both these models have been implemented to evaluate potential vaccines [82, 119, 161, 162] and in the case of AHF, immunotherapy [163], and therapeutics [164, 165]. AHF model has been fairly well characterized [127–130], but less is known concerning pathogenesis of LASV. Unlike the macaque models, only one pathogenic strain has been evaluated for both AHF (JUNV, XJ strain) and LF (LASV, Josiah). Marmosets are not hypersusceptible to arenavirus infection as virulence was not detected for Tacaribe virus, a NW arenaviruses not pathogenic in humans [161]. Additionally, attenuated strains of JUNV (XJ44) and LASV (ML29) were not virulent in marmosets [119, 162].

In line with both human and macaque infection, marmosets exposed with 1000 PFU of Lassa virus (Josiah) exhibit signs of infection on day 8 [118]. Similar to the macaque models, initial signs of disease are nonspecific, such as weight loss, anorexia, and mild fever. Viremia can be detected by day 8 and reach high levels before euthanasia, approximately 3 weeks after infection [118, 119]. There were no alterations in white or red cell counts, but platelets decreased over the course of disease. Indications of hepatocellular damage, such as increases in AST, ALT, and ALP (later in disease) and a decrease in albumin, were also noted. Reminiscent of human disease (and the macaque model) viral burden in the liver was high, but histological changes were mild. A decrease in liver-associated lymphocytes, coupled with a decrease in MHC II expression suggests a LASV-specific immunosuppressive effect [118]. Other manifestations, such as adrenal necrosis, also coincide with human disease. The interstitial pneumonia described in marmosets and macaques has not been described in humans.

Like the LF model, JUNV infection in the common marmoset shares many disease features common to humans and experimentally infected macaques. Subcutaneous exposure with 1,000 TCID_50_s of JUNV strain XJ produces signs of illness such as anorexia, decrease in weight, leukopenia, thrombocytopenia, viremia, neurological illness (tremors), and hemorrhage of the gums, pharynx, and esophagus abdominal cavity, in addition to death [127–130]. A profile of coagulation parameters and complement activity were also reported but are inconsistent with known human disease and other NHP model systems [129]. However, in general disease in this model is similar to human disease.

Other potential arenavirus NHP models have been explored. These have included the use of African green monkeys (*Chlorocebus aethiops*) and tamarins (*Saguinus geoffroyi)* for BHF studies, and African green monkeys and hamadryas baboons for LF studies [96, 166, 167]. Subcutaneous inoculation of African green monkeys with 1,000 PFU of the MACV strain Carvallo produced an abbreviated incubation period and more severe disease relative to macaques [167]. Similar to the macaque, signs of infection include conjunctivitis, anorexia, fever, hemorrhage, viremia, decrease in neutrophils and lymphocytes, and neurological manifestations [167, 168]. Tamarins exposed to MACV exhibit anorexia, tremors, shock, viremia, and succumb 8–20 days after exposure [96]. African green monkeys infected with LASV results in a disease similar to that observed in as macaques [6]. LF-like disease can be also be produced in hamadryas baboons after aerosol exposure or i.m. injection with LASV and is characterized by fever, hemorrhage, and viremia [166]. The baboon model has been used to evaluate Virasol (ribavirin) [169] and an inactivated vaccine [170]. LASV infection of squirrel and capuchin monkeys fails to produce disease, demonstrating that not all NHPs are susceptible to arenavirus infection [6]. Given the vast amount of data produced regarding arenavirus infection in macaques, this system is by far the preferred animal model for the study of arenavirus pathogenesis and MCM development. At least one study suggests that cynomolgus macaques are more susceptible to lethal disease [157], but this would require a more thorough investigation. Because of their emerging prominence in infectious disease animal research due to their smaller size and primate immune system [171], marmosets may offer a valuable alternative to using other NHPs in the study of arenavirus disease and MCM development. However, arenavirus studies in this model are much more limited and further investigation is warranted to determine if, like macaques, disease in marmosets is analogous to that observed in humans.

### 2.6. Development of Low Containment Arenavirus Animal Models through Adaptation of Pichinde Virus to Hamsters and Guinea Pigs

Due to complexities of BSL4 working environments (logistics, cost, and space) several groups have developed low containment animal models (BSL2 and 3) to study arenavirus HF disease. These models exploit arenaviruses avirulent in humans and have included use of Tacaribe virus in IFN knockout animals, Pirital virus in hamsters and Pichinde virus (PICV) in hamsters and guinea pigs. These models have been reviewed elsewhere [5]. Of these, PICV animal models are worth expanded discussion as they offer not only reasonable models of arenavirus infection in immune-competent adult animals, but also serve as a primer for the adaptation of arenaviruses to animals.

PICV is a NW arenavirus (clade A) first isolated in Columbia in 1970 from its rodent carrier* Oryzomys albigularis* by Trapido and Sanmartín [172]. PICV is avirulent in humans and is a BSL2 agent. Buchmeier and Rawls explored the lethality of PICV strain An3739 in inbred (MHA) and outbred (LVA) hamsters (*Mesocricetus auratus*) [142]. In that study, strain An3739 only caused lethal disease in inbred MHA adult hamsters, whereas infection in outbred LVA adult hamsters did not result in significant lethality unless animals were treated with cyclophosphamide (150 mg). Infection of inbred hamsters with 500 PFU by the s.c. route produces a viremia that continues to rise until death that generally occurs as early as day 7 and as late as day 21, depending on infectious dose. The authors found that PICV strain An3739 is lethal in outbred LVA hamsters younger than day 9 (neonates). Subsequent to this study, Smee et al. explored the use of another strain of PICV, strain An4763, which they found to be lethal in older (>3 week old) outbred (LVA) hamsters [143]. In this model, hamsters developed disease signs including viremia and increased liver enzymes. Virus could also be detected in the lung, liver, and spleen with titers that increased over the course of disease. Infections with doses ranging from 10^2^–10^4^ PFU/animal were uniformly lethal with mean time to death ranging from 6 to 10 days. The mechanism behind attenuation of An3739 in outbred hamsters is unclear but may involve virus passage history in newborn hamster brains. Inbred MHA, but not outbred LVA hamsters, are deficient at producing IL-2 and this cytokine may play a role in host susceptibility to infection [173]. Several studies have exploited the PICV strain An4763 outbred hamster model to investigate the protective efficacy of antivirals targeting arenaviruses, including interferon alfacon-1 [174]. Inbred MHA hamsters appear to be no longer available for further study. Outbred LVA hamsters are more commonly called Golden Syrian hamsters and are available from a variety of venders.

A guinea pig adapted PICV animal model was developed by Jahrling et al. [144]. The same strain used in outbred hamsters, PICV strain An4763, is not lethal in Strain 13 guinea pigs; however 18 continual passages of virus in these animals produced a clone, termed P18, which was lethal. Adaptation involved genetic factors present on both the L and S gene segments are involved in virulence [175]. Strain 13 guinea pigs are highly susceptible to lethal disease by P18 when the inoculum is >3 PFU (s.c. injection) with all animals succumbing to disease by day 19. Hartley guinea pigs are refractory to lethal disease except at high infectious doses (3,000 PFU) but even then only 43% of the animals succumbed to infection [144]. The guinea pig model, despite limited availability of strain 13 animals, could provide valuable insight into pathogenesis, as well as insights into protective immune responses necessary for protection against primary and secondary arenavirus infections. As mentioned above, the methodology for adaptation of PICV to guinea pigs could be emulated with LUJV and LASV to generate strains adapted to Hartley guinea pigs.

Both the hamster and guinea pig PICV infection models are surrogate models for LASV infection. This may be owed to similar receptor usage as NW arenavirus of clade A usurp *α*-dystroglycan for cellular entry, whereas clade B NW arenavirus bind transferrin receptor [8]. Whether guinea pigs or hamsters make a better surrogate LASV model has been debated. Groups supporting a focus on guinea pigs argue that this model is superior given that strain 13 guinea is the small animal model for LASV [144]. Others have suggested that the smaller size of hamsters provides an advantage in the initial screening of MCMs because less product is required due to animal weight [143].

## 3. Hantavirus Animal Models

### 3.1. Mice

Currently, there are no good small animal models that faithfully recapitulate human HFRS disease. Early pathogenicity study examined infection in suckling mice [176–180] and rats [181, 182]. Infection of newborn mice with HTNV though multiple routes including i.c., i.p., i.m., and s.c. results in lethal disease with widespread viral dissemination characterized by histologic lesions in the brain (diffuse meningoencephalitis with bilaterally symmetrical thalamic necrosis), liver (pericholangiohepatitis), lung (pneumonitis), and spleen (lymphoid hyperplasia) [178]. The age of the mice is critical to the disease outcome. 100% lethality occurs only in three-day-old mice but lethality decreases rapidly with age and is only 50% lethal in one-week-old mice and not lethal in two-week-old animals. Similarly, sublethal infection of newborn BALB/c mice [183] with HTNV results in an asymptomatic persistent infection consistent with that seen in the natural rodent host,* Apodemus agrarius*. While infection of newborn mice with hantaviruses does represent a disease model, it fails to recapitulate many of the characteristics of human hantavirus disease. Perhaps most concerning is that infection of newborn mice results in a lethal neurologic disease leading to hind limb paralysis and death uncharacteristic of human HFRS or HPS and evidence of microvascular edema is lacking. Commonly used animal models for hantavirus are listed in Table 3 and viral strains are listed in Table 4.

The amelioration of disease as newborn mice age would suggest that adult laboratory mice would also fail to develop disease. Wichmann et al. demonstrated that i.p. Hantaan virus infection of 8-week-old C57BL/6, SJL/J, BALB/c, and AKR/J mice (listed in order of decreasing susceptibility) with 10^5^ PFU of HTNV strain 76–118 resulted in a lethal neurological disease similar to that in suckling mice [215]. However, more recent studies by Klingstrom and colleagues concluded that infection of BALB/c and C57BL/6 strains with HTNV and/or three other strains of HFRS-associated hantaviruses, Puumala virus (PUUV), Dobrava virus (DOBV), and Saaremaa virus (SAAV), did not cause disease in mice [193]. Generally, murine infection induces T cell [216, 217] and humoral immune responses against HTNV, DOBV, and SAAV and wild-type PUUV [193]. Interestingly, mice challenged with cell culture-passaged PUUV exhibited lower IgG anti-N titers compared to wt PUUV or, in some cases, failed to seroconvert altogether [193]. Why the same strain of virus, administered by the same route to the same species of mouse, would result in dramatically different outcomes has yet to be explained but the results may suggest that the age/maturity of the mouse may be a factor contributing to the outcome (persistence versus clearance versus disease) following hantavirus infection. To date, we are unaware of attempts to infect adult laboratory strains of mice with HPS-associated hantaviruses. Overall, given the disparity between human disease and infection laboratory mice, this system is inadequate for the study of hantavirus pathogenesis.

Despite being poor pathogenesis (natural disease) models, mice are used extensively in evaluation of vaccine immunogenicity and protective efficacy. BALB/c mice [196, 218–236] have been the predominant model to study hantavirus vaccines but others including C57BL/6 [215, 227, 229, 237–246] and NMRI [247] mice have also been employed to demonstrate vaccine efficacy against HTNV [215, 218, 219, 223, 225], PUUV [221, 222, 232, 236, 247], SEOV [196, 232], DOBV [227, 248], ANDV [235], and SNV [220, 232, 249, 250]. Inactivated HTNV virus, one of the earliest hantavirus vaccines, was initially tested in the murine model and was found to produce protective cellular and humoral responses, including neutralizing antibody protection [218, 219, 223, 240, 241, 244–246]. Subsequently subunit DNA vaccines and virus vector vaccines, including vaccinia virus, lentivirus, adenovirus among others, targeting nucleocapsid, and glycoproteins were also tested in murine models and found to induce broad seroconversion, including robust neutralizing antibody titers and a mixture of T_h_1 and T_h_2 T cell responses capable of protecting mice from hantavirus infection [196, 215, 220–222, 229, 230, 235, 246, 249, 251]. Virus-like particles (VLPs) vaccines [226, 241] have also been tested in this model. Each subunit or VLPs vaccines were immunogenic, producing cellular and humoral immune responses equal to or superior to those afforded by inactivated whole virus vaccines in mice.

### 3.2. Immunocompromised Mice

Experimental infection of immunocompromised severe combined immunodeficient (SCID) mice and nude mice, both deficient in adaptive immune responses, have shown much greater dissemination of HTNV and SEOV to multiple tissues compared to immunocompetent BALB/c mice [252] with the highest concentration of virus being found in the brain and kidneys. However, viral distribution was more substantial in SCID mice and only SCID mice infected with HTNV or SEOV developed a lethal wasting disease, characterized by ruffled fur and weight loss, approximately 5 weeks after challenge rather than the neurological disease seen in newborn mice. Viral antigen was not restricted to endothelial cells but was also found in parenchyma cells in the kidney, lung, liver, heart, and brain [252, 253]. This model has been used in subsequent experiments to investigate the role of various immune cell subsets in hantavirus disease pathogenesis. Up to 14 days after infection, adoptive transfer of splenic T and B cells from donor mice into SCID mice protected SCID mice from disease but adoptive transfer after 14 days after infection failed to protect SCID mice [254]. Adoptive transfer of splenic T and B cells was observed to increase levels of serum blood urea nitrogen (BUN) levels [252], consistent with abnormal kidney function and human HTNV infection but gross kidney pathology was not observed. Moreover, the presence of T and B cells resulted in an increase in pulmonary edema in the lungs which can be partially reversed by depleting neutrophils during the acute disease phase of HTNV infection [253] but did not alter the mean time-to-death.

### 3.3. Hamsters

Infection of outbred Syrian hamsters (*Mesocricetus auratus*) with HTNV, SEOV, DOBV, or PUUV results in an asymptomatic, disseminated infection (Table 3) [188, 196, 255]. These infection models have been used extensively to gauge neutralizing antibody efficacy in passive transfer experiments [46, 188, 189, 196, 201, 256, 257]. Similarly, in immunocompetent hamsters, SNV, a causative agent of HPS, results in an asymptomatic infection with undetectable viremia [197, 199]. Even infection with hamster-adapted SNV (achieved by 20 serial passages of SNV-infected deer mice lung homogenate in hamsters) fails to cause an HPS-like disease in immunocompetent hamsters despite increased viral dissemination and detectable viremia [200]. By comparison, hamsters infected with the HPS-associated hantaviruses ANDV or Maporal virus (MAPV) develop disease that is remarkably similar to HPS pathogenesis in humans as first described by Hooper et al. in 2001 [197]. After an incubation period ranging from 9 to 13 days, MAPV infection of hamsters results in marked lung and liver pathology, production of virus-specific IgG, and approximately 30% lethality [205]. ANDV infection of hamsters results in a similar incubation period which varies upon route of infection. Consequently, infection with ANDV in hamsters results in a disease onset characterized by severe dyspnea, pulmonary edema, and fluid in the pleural cavity [197]. Thickening of alveolar septal walls can be observed in the lung by day six, with increased damage over time resulting in severe pulmonary edema, mononuclear cell infiltration, and hemorrhage 14 days after infection [199]. Variable levels of hepatitis and hepatocellular necrosis were observed in livers of ANDV-infected hamsters. Infection of hamsters with 2,000 PFU of ANDV, regardless of route (s.c., i.m., i.n., and intragastric [i.g.]), results in a 100% lethal animal model [62]. Similar to the kinetics of neutralizing antibodies in severe human cases of HPS, the presence of neutralizing antibodies appears late in the course of disease and is insufficient to protect hamsters from lethal disease [197]. This model resembles human disease emulating some of the more salient features of HPS including pulmonary edema and acute respiratory distress. As a result it is considered the gold-standard of small animal hantavirus disease models.

More recently, Hardcastle et al. have demonstrated that Laguna Negra virus infection of Turkish hamsters (*Mesocricetus brandti*) causes a lethal vascular leak syndrome similar to HPS in humans and HPS-like disease caused by ANDV in Syrian hamsters [258]. Indeed, many of the clinical features ascribed to human HPS, rapid onset of the cardiopulmonary phase of disease and histopathological changes in the lung, are reproduced in the Turkish hamster model of HPS. In this model, intraperitoneal challenge with 100 PFU LNV results in signs of clinical disease approximately 12–16 days after challenge and a 43% fatality rate. Compared to the HPS-like disease found in Syrian hamsters infected with ANDV, the onset of lung pathology can be detected in both models as early as 5-6 days after challenge with an increase in severity peaking 10–12 days after challenge; the temporal differences in disease pathogenesis is most likely due to differences in challenge dose and route. Both models result in disease characterized by a rapid onset of severe dyspnea coinciding with rapidly progressing lung pathology, multifocal to diffuse interstitial pneumonia with alveolar edema, and hemorrhage and with a corresponding presence of viral antigen associated with endothelial cells.

Perhaps the most significant differences between the Syrian hamster and Turkish hamster HPS models are the discordance in fatality rates and the observation that Turkish hamsters infected with LNV progressively lose weight as disease progresses whereas the weight of Syrian hamsters infected with ANDV remains steady or increases. The authors also suggest that Turkish hamsters infected with LNV display a panleukopenia compared to leukocytosis seen in Syrian hamsters after ANDV challenge. In general, infection of Syrian hamsters with ANDV does result in leukocytosis from the time of challenge to the onset of visible symptoms (dyspnea, staggered gait); however, a pronounced leukopenia in both peripheral blood and lung tissue is observed between the onset of visible symptoms and severe disease/humane endpoints [199, 259].

The 43% fatality rate is reminiscent of MAPV infection of Syrian hamsters but in contrast to MAPV [205], which is not known to be a human pathogen, LNV has been associated with an outbreak of human disease in Paraguay in 1997. Moreover, the lower fatality rate in hamsters appears to more accurately reflect the fatality rate associated with human HPS. It is not known whether a higher LNV challenge dose would result in uniform disease or if LNV is able to cause disease in hamsters when administered by other routes of infection such as intramuscular or intranasal. Worth noting is that among the 57% of hamsters designated as survivors, none of these animals exhibited mild or transient clinical signs to suggest that these survivors recovered from illness. All animals infected with LNV did seroconvert potentially suggesting that slight genetic differences between hamsters made the difference between viral clearance and disease pathogenesis. Still, comparisons between ANDV and SNV infection of immunocompetent and immunocompromised Syrian hamsters, and the Turkish hamster/LNV lethal disease model could provide investigators with an important tool to use to understand why different hantaviruses are associated with differing degrees of mortality and morbidity in similar hosts.

The ANDV hamster model has been used to evaluate several viral vector, DNA-based, and virus like particle (VLP) vaccines. Both adenovirus and vesicular stomatitis virus (VSV) vectors expressing hantavirus proteins provided sterile immunity in hamsters against an otherwise lethal ANDV challenge [235, 260, 261]. An ANDV DNA-based vaccine targeting glycoproteins (M-segment) was not immunogenic or protective in hamsters [262]. However, a similar DNA vaccine targeting PUUV was immunogenic in hamsters, producing neutralizing antibodies when delivered by identical means [263] and protects hamsters from ANDV challenge [264]. The ANDV/hamster model has been used to test protective efficacy of neutralizing antibodies produced using DNA vaccination of NHPs, rabbits, ducks, geese, and humanized bovines and in each case protected animals 5 days after i.m. ANDV challenge and up to 8 days after i.n. challenge [62, 262, 265–267]. However, passive protection is not possible after onset of viremia. A similar study performed by the Hantavirus Study Group in Chile found that treatment of patients with human ANDV convalescent serum resulted in a borderline statistically significant decrease in HCPS case fatality rates for a cohort of 32 individuals between 2008 and 2012 [268]. The lack of a greater effect compared to the results obtained in hamsters is likely due to the progression of disease in humans prior to treatment with convalescent serum. Patients admitted to the study already exhibited mild/moderate to severe disease and had confirmed ANDV infection based on anti-ANDV IgM titers and/or positive ANDV RT-PCR. By comparison, treatment of hamsters with human convalescent serum is only effective if given prior to viremia (day 5 after challenge) based on positive ANDV RT-PCR and/or plaque assay. When hamsters are treated after viremia has occurred (day 8 after challenge), human convalescent serum fails to protect against disease. Interestingly, the factor that correlated most with a positive clinical outcome (i.e., survival) was disease severity at the time patients were admitted to hospital. A greater number of patients admitted with milder disease had a positive clinical outcome compared to patients admitted with more severe disease whether or not those patients received convalescent serum treatments. Patients admitted with confirmed cases of HPS are routinely given supportive care which can include careful monitoring and regulation of fluids, intravenous treatment with vasopressin and dobutamine, supplemental oxygen, and ECMO, if necessary [269, 270]. These supportive treatments at least partially explain the greater survival rates compared to hamsters which can be difficult or impractical to administer to hamsters.

In addition to testing immune-based MCMs, two nucleoside analogue antivirals, ribavirin and favipiravir (T-705), have shown efficacy when delivered as postexposure prophylaxis to ANDV-challenged hamsters [271–273]. Treatment with these drugs, as with passive transfer of neutralizing antibodies, is only efficacious in hamsters prior to the onset of viremia.

The hamster model has also been used to examine the pathogenesis of hantaviruses. Using such a transcriptional profiling approach, Safronetz et al. analyzed the kinetic expression of numerous pro- and anti-inflammatory cytokine genes following intranasal challenge of hamsters with 200 FFU ANDV. Their results demonstrated a strong upregulation of both pro- and anti-inflammatory T_H_1 and T_H_2 genes beginning 7-8 days after challenge that was primarily localized to the lung, despite readily detectable hantavirus antigen associated with endothelial cells in a majority of hamster tissues [198]. Also noted was the relative static or decreased expression of regulatory FoxP3 and TGF*β* genes following ANDV infection. This apparent absence of a regulatory counterbalance to the inflammatory immune response to ANDV infection, found almost exclusively in the lung, combined with the correlation between inflammatory cytokine gene expression and the onset of lung pathology, has furthered speculation that, like human disease, HPS in hamsters is caused by immunopathological mechanisms. The role that T cells might play in the HPS-like disease in Syrian hamsters is less obvious. Similar to the pattern of proinflammatory cytokine gene expression described by Safronetz et al., we demonstrated that activated T cells first begin to infiltrate in the lungs approximately 5 days after 2,000 PFU intramuscular ANDV challenge correlating with the first signs of lung pathology [199, 259]. Consistent with the increased numbers of T cells in the lungs of severe human HPS, peak accumulation of T cells in the lungs of hamsters is between 8 and 10 days after challenge which in many cases is just prior to terminal disease. While the percentage of activated T cells in the lungs of hamsters is relatively similar to the percentage of SNV-specific T cells reported for human HPS by Kilpatrick et al. [73], at this time it is impossible to determine the percentage of hamster T cells specific for defined hantavirus glycoprotein and nucleocapsid epitope due to the lack of hamster-specific reagents. While these results would suggest a strong correlation between severe lung pathology and T cell accumulation in the lung, the depletion of T cells in hamsters prior to ANDV infection [274] or prior to acute disease [259] in hamsters fails to protect hamsters and prevent disease.

One interesting qualitative difference between the T cell responses to hantavirus infection in hamsters and humans is that human T cell responses trend towards a skewed CD4^+^ T cell to CD8^+^ T cell ratio in favor of the CD8^+^ compartment [275] where as in hamsters, the predominant population is of the CD4^+^ variety [259]. The vascular endothelium remains intact and continuous during hantavirus infection of both humans and hamsters arguing against immunopathology mediated by perforin and granzyme typically associated with CD8^+^ T cell response but in favor of a cytokine-driven mechanism orchestrated by potentially numerous cell types. Moreover, experiments by Gupta et al. [276] have shown that ANDV nucleocapsid protein inhibits the enzymatic activity of granzyme B and caspase 3 suggesting a mechanism by which hantavirus infection may render endothelial cells resistant to CD8^+^ T cell and NK cell cytotoxic responses. Still, it has been suggested by some that hamsters represent a poor model to study hantavirus immunopathology because the level of immune activation is not as robust as that seen in humans and nonhuman primates [202]; however, until a more diverse set of hamster specific reagents is developed, it is difficult to accurately estimate the breadth of the immune response to hantavirus infection in hamsters. Whether the disease mechanism in humans, nonhuman primates, and hamsters is the same has yet to be determined. Alternatively, disease in the absence of a robust immune response may suggest a mechanism of disease other than one that is immune mediated.

Despite the overall importance of the hamster model to hantavirus pathogenesis studies, the characterization of the immune response to hantavirus infection in hamsters remains in its infancy. The dearth of immunological reagents for the hamster, when compared to the overwhelming abundance of mouse, rat, human, and nonhuman primate reagents, has made a direct comparison of cellular immunological findings between HPS in humans, and HPS-like disease in hamsters, difficult. However, several studies have begun to identify multiple reagents that can be used in the hamster system. We have produced a list of available reagents previously shown to react with the hamster immune system (Supp Table 1, in Supplementary Material available online at http://dx.doi.org/10.1155/2015/793257). In addition to these reagents, the recent sequencing, annotation, and analysis of the Syrian hamster transcriptome [277] should also facilitate the expansion of a new generation of resources to study the hamster model.

### 3.4. Immunosuppressed Hamster Model

Using a combination of immunosuppressive drugs, dexamethasone and cyclophosphamide, hamsters infected with SNV develop disease that accurately mimics both HPS disease in humans and ANDV infection of hamsters [201]. Similar to ANDV infection of immunocompetent hamsters, SNV infection in immunosuppressed hamsters (2,000 PFU by the i.m. route) results in a 100% lethal model with LD50 as low as 2 PFU (Table 3). Similar levels of SNV viral genome were amplified from lung tissue harvested 28 days after challenge in all surviving hamsters. In this immunosuppressed model, there is increased viral dissemination and pathology of the lung showing marked inflammation and edema within the alveolar septa of SNV-infected animals. The ability to prevent HPS disease with neutralizing antibody treatment highlights the specificity of this model and its utility as an alternative model for the testing of MCMs.

### 3.5. Persistent Hosts

Similar to arenaviruses, each hantavirus is associated with a specific mammalian host species in a specific geographical area [278] which remains persistently infected for life, continually shedding virus. While many small mammals, including rodents (mice, rats, and voles), insectivores (shrews and moles), and bats, serve as reservoirs for hantaviruses [279, 280], only rodent-borne hantaviruses are known to be associated with human disease. The University of New Mexico has developed a deer mouse (*Peromyscus maniculatus*) containment laboratory to exclusively study SNV infection in its natural host [203]. In this model, SNV is widely disseminated throughout the host without pathology, with the transition from acute to persistent infection occurring 60–90 days after infection (Table 3) [203, 281]. A sentinel study that examined experimentally infected deer mice with SNV or ANDV began to shed light on how SNV establishes a persistent infection in its host. SNV infection in deer mice resulted in reduced immune gene expression and virus-specific antibody response, whereas ANDV infection resulted in high levels of immune gene expression and antibody responses [282, 283]. ANDV is cleared from the animal rapidly, whereas SNV establishes a long lasting persistent infection.

Two other persistent infection models have been described for HFRS hantaviruses. This includes PUUV infection of its natural host the bank vole (*Myodes glareolus*) and SEOV infection of the Norwegian rat (*Rattus norvegicus*) (Table 3) [191, 284]. Similar to SNV infection of deer mice, suckling and weanling bank voles experimentally infected with PUUV have disseminated infection without pathology [191]. Experimental infection of the Norwegian rat with SEOV has shown reduced proinflammatory cytokine and chemokine expression in the lung with elevated regulatory responses which may contribute to persistence [285]. This same laboratory has also identified genes differentially expressed in male versus female rats, with implications on the transmissibility of the hantavirus [286, 287]. These model systems show that hantavirus infection in its host rodent has reduced immune expression when compared to heterologous hantaviruses, contributing to persistence [288]. Furthermore, these models will undoubtedly shed greater light on how these viruses establish an asymptomatic, or limited symptom, infection in one host, and catastrophic disease in another.

### 3.6. NHP Models

Initial attempts to create a hantavirus nonhuman primate (NHP) disease model were largely unsuccessful. Cynomolgus macaques infected with either Prospect Hill virus or ANDV failed to develop clinical disease (Table 3) [206, 289]. Infection of cynomolgus macaques with PUUV results in a disease similar to nephropathia epidemica in humans; the monkeys were lethargic with mild proteinuria and histopathology noted in medullary tubular cells of the kidneys [192]. In-depth analysis of cytokines and other mediators produced in response to PUUV infection (i.e., IL-6, IL-10, TNF-*α*, and nitric oxide), as well as pathological studies show consistency with human PUUV infections [290, 291]. Passive transfer of neutralizing antibodies can lessen the severity of HFRS disease [292] or provide sterilizing protection [293].

Recently, the first NHP HPS disease model has been described (Table 3) [202]. In this study, rhesus macaques were infected with SNV passaged only in deer mice by the i.m. route. The infectious dose was 6 × 10^6^ RT-PCR values and did not involve viral purification but rather administration of homogenized tissue. Hematology results demonstrate thrombocytopenia and leukocytosis, with severe pulmonary edema, that is, characteristic of HPS infection in humans. As with humans, immune responses were especially prevalent in the lung in addition to pathological abnormalities. This new model will provide an alternative testing platform for HPS vaccines and therapeutics as well as a system for the study of viral pathogenesis. However, the ability to produce sufficient virus exclusively using deer mice may present a logistical hurdle, which potentially may be surmounted by growing SVN in deer mouse cells. Nevertheless, this model will clearly be a valuable tool for emulating HPS human disease.

## 4. Important Considerations

### 4.1. Routes of Infection

Both hantaviruses and arenaviruses infect humans through exposure to rodent excreta and/or secreta (saliva and urine). Humans are infected from fomites through the upper and lower respiratory tract (aerosols), through skin abrasions and also ingestion. The most efficient means of biological weapons dispersal is thought to be the aerosol route [294]. Arenavirus can cause lethal infection in both guinea pigs and NHPs when delivered by a multitude of routes including i.n., aerosols, i.m., s.c, i.p, and orally (Table 2). Studies with PICV suggest that the s.c. route may be less lethal versus the i.p. route [143]. Andes virus has been shown to infect hamsters by i.n., i.m., oral, and s.c. routes (Table 3). The route of infection can impact disease kinetics and time to death. Andes virus by i.n infection is slower versus i.m. with a mean day to death of 17 and 13 days, respectively [62]. Infection by the oral route can limit the virulence of JUNV and smaller percentages (40–60% versus 100% lethality) of animals succumb to infection versus parenteral routes [295]. This is likely due to inactivation of the virus at acidic pH upon entering the alimentary canal. ANDV infection by oral gavage also results in death and lethality remains at 100% by this route [62] although we are not aware of any cases of human disease caused by the ingestion of hantaviruses. Ideally, a demonstration that a MCM can protect against multiple routes is most desirable with at least one being a natural route of exposure (i.e., mucosal routes). However, to demonstrate the effectiveness of an MCM against a biological weapons threat, there is essentially a prerequisite to show protection against an aerosol challenge.

### 4.2. Heterogeneity among Virus Strains

The choice of arenavirus strain is of critical importance to arenavirus animal models. Different isolates of JUNV generally cause two different disease patterns in humans (hemorrhagic or neurologic) and this has also been observed in NHPs and to a lesser extent guinea pigs [81]. In addition, various strains of arenaviruses can cause varying degrees of lethality ranging from 0% to 100%. Therefore great care must be taken to address the experimental question being tested. Strains causing 100% lethality may be ideally suited to test MCMs, assuming this would be the most stringent test of efficacy. In contrast, studies seeking identification of biomarkers of virulence and virus/hosts factors contributing to disease severity would be best served using a strain that causes reduced lethality, such as strain JUNV MC2 [85]. Similarly a newly identified strain of LASV (Soromba-R) does not cause 100% lethality in guinea pigs or NHPs [101] and would be useful in the identification host factors contributing to lethal and nonlethal outcomes.

Use of the same strain of virus in both rodent and NHPs models may or may not be possible depending on virus species. LASV strain Josiah and Z-132 are both uniformly lethal in guinea pigs and NHPs, with Josiah being the prototypical strain used in the study of LF. Recent data using MACV strain Chicava indicated that this strain is uniformly lethal in both guinea pigs and NHPs [97]. However, JUNV studies may require use of different strains as the prototypical JUNV Romero strain used in the guinea pig model while >90% lethal in guinea pigs is not lethal in NHPs. Similarly, SNV propagated in Vero E6 cells does not cause disease in hamsters and NHPs, but SNV isolated from the lungs of deer mice used to infect NHPs is 70% lethal [197, 202]. The use of identical strains for NHP and rodent models may be of importance in pathways to licensure of MCMs. However, use of different strains in different models may provide greater insight as to the protective efficacy of a given MCM and its ability to thwart a heterologous virus that may be encountered in nature.

There are no serotypes of arenavirus and hantavirus species* per se*. However there are studies clearly demonstrating differences in susceptibility to neutralizing antibodies for arenaviruses. For example, Candurra et al. found that hyperimmune rabbit serum was most potent at neutralizing homologous versus heterologous viruses and values against the latter were up to 22-fold lower [85]. Strains of LASV taken from different regions are also antigenically unique with varying impacts on neutralization titers [102]. Sierra Leone and Liberian isolates are most homologous, with Nigerian counterparts being less so. Therefore considerations must be taken in the study of MCM where antigen homology is of concern, such as vaccine and antibody-based protection studies. Accordingly, a finding that a MCM targeting strains from one region should be tested to confirm it also protects against heterologous strains.

Passage histories of arenaviruses and hantaviruses can have a dramatic impacts on disease course in particular animal models. SNV passed in cell culture loses virulence for NHPs; however, virus passaged exclusively in deer mice (the natural host) cause severe disease in infected animals [202]. Genetic analysis showed only a single amino acid difference in the polymerase coding region between SNV isolated from NHP to Vero-passaged SNV. How this impacts virulence remains enigmatic. Arenaviruses have a history of losing virulence during passaging. For example, MACV strain Carvallo once reported to cause a lethal infection in adult guinea pigs was no longer pathogenic in this model (Golden, J. W., manuscript forthcoming and 97). Similarly, passages of JUNV strain XJ, once reported to be 100% lethal in guinea pigs [295, 296] were found to be attenuated in Hartley and strain 13 animals [83]. The molecular determinants of attenuation are only starting to be investigated and for arenaviruses may involve subtle changes, including at least one in the glycoproteins, GP2 [121]. Fortuitously, a reverse genetic system has been developed for arenaviruses that will circumvent problems associated with passage attenuation and greatly aid in strain curation/preservation, as well as providing fundamental insight into virus biology. Several studies have genetically reconstituted arenavirus species for* in vitro* and* in vivo* studies including, JUNV, MACV, and LUJV [103, 135, 297]. The ability to maintain strain homology may be critical in the development and evaluation of MCM against arenaviruses. In this regard, the reverse genetic system will be extremely useful in the harmonization of arenavirus species between different laboratories. Unfortunately no reverse genetic system yet exists for hantaviruses.

### 4.3. Animal Rule

The Kefauver-Harris US Federal Drug Administration (FDA) amendment in the 1960s added the requirement that new drugs prove that they are not only safe, but efficacious. The number of human cases of PUUV and LASV infections (as well as a few others) are likely sufficient enough for human phase trials to provide proof of efficacy for MCMs. However, the relatively limited number human infections caused by some arenaviruses (e.g., MACV, GTOV, and LUJV) and hantaviruses (e.g., HPS-causing hantaviruses) means that the feasibility of conducting clinical trials to provide proof of efficacy for MCMs will be problematic. Because exposing individuals to agents capable of causing severe disease is unethical, the FDA has provided a pathway for the approval of drugs (21 CFR 314.600) or biological products (21 CFR 601.90) under circumstances where human efficacy studies are not feasible. Because animal modeling systems are required to provide proof of efficacy for a given intervention, these regulations have commonly been referred to as “The Animal Rule” [298]. Suitability of the model will depend on many factors the most critical of which is the recapitulation of human disease. Because no model will likely be a complete transcript of natural human disease, it is probable that multiple models will be required to satisfy the rule. Choosing animal models to evaluate potential countermeasures is no trivial task, but the FDA has updated their guidance document to help clarify the criteria required for approval under the Animal Rule. Given the complexity of this issue it is difficult to determine which animal models discussed in this review will be acceptable for the animal rule. Those developing MCMs against arenaviruses and/or hantaviruses would be encouraged to enter into a dialogue with the FDA as early in the developmental pathway as possible so a valid pathway forward can be determined.

## 5. Summary

One method to mitigate the threat of rodent-borne viruses has been rodent control. During a MACV outbreak in Bolivia, trapping of* Calomys callosus* proved to be somewhat successful [299]. However, attempts in Africa with the far more wide-spread* Mastomys natalensis* have generally failed [300]. Thus, the best hope for mitigating disease from these animals is the development of MCMs. The gold standard small animal models for arenaviruses and hantaviruses are the guinea pigs and hamsters, respectively. The faithful recapitulation of arenavirus disease in both OW and NW NHPs makes them the gold standard large animals for MCM development and the study of pathogenesis. Whereas previously hantavirus NHP models were essentially nonexistent for HPS-causing viruses, the recent finding that SNV produced in deer mice produces acute disease in infected macaques opens new opportunities for the testing of candidate countermeasures against HPS as well as factors that contribute to pathology. It may also help guide the development of similar models with other Hantavirus species. The requirement for SNV to be produced in deer mice and not passaged in cell-culture to be virulent in NHPs highlights the importance impact strain heterogeneity can have on an animal model.

It is clear from published works that there is a trend to focus on highly lethal models of infection. Logically this is the most stringent way to evaluate a given MCM. However, it is important to point out that studies in animals for which lethality is well below 100% may lead to clues about viral and host factors critical for acute disease. The best example is perhaps LASV in Hartley guinea pigs for which lethality is between 30 and 70%. In addition, the development of a LASV strain that is 100% lethal in Hartley guinea pigs may also yield critical insight into virus genetic factors involved in virulence. These types of studies have shed light on the importance of virulence factors for Ebola virus, where mutations in VP24 play virus spread in guinea pigs [301].

In 2012, there was an outbreak of HPS in Yosemite National Park caused by SNV infected deer mice living in the walls of tents used by tourists [302]. Furthermore, about every 3 years novel arenaviruses capable of causing acute human disease emerge [8]. The most recent of these was the emergence of CHPV and LUJV in South America and Africa, respectively [15, 16]. These serve as reminders that these rodent-borne viruses continually emerge and reemerge and are likely to grow in number due to human encroachment on rodent habitats. Thus the continued study of these viruses in both models of acute disease and in their natural hosts is of critical importance and will help guide future MCM development.

## Supplementary Material

Hamsters represent an important small animal model to study a variety of fields including metabolism, carcinogenesis/tumorigenesis, inflammation and infection and disease. However, hamster models are often overlooked by many investigators, resulting in a lower popularity when compared to mouse and rat models. In some cases, as with New World hantaviruses, hamsters represent the only known small animal model of human disease caused by these viruses, elevating their importance as a tool to understand human disease pathogenesis. Still, the dearth of hamster-specific reagents to study many aspects of disease pathogenesis and immunological processes, when compared to the overwhelming abundance of mouse, rat, human, and nonhuman primate reagents, has made a direct comparison of human disease and hamster disease, difficult. In rare instances, commercial vendors such as MyBioSource.com have begun selling hamster-specific reagents. Unfortunately, this is more the exception than the rule as most reagents used to study hamsters are cross-reactive mouse, rat and human reagents that have been identified by trial and error. In the following table, we have attempted to consolidate those, predominantly immunologically related, reagents that we and others have used in the hamster model.

## Figures and Tables

**Table 1:** Prototypical human pathogenic arenavirus strains and their origin.

Virus	Strain	Origin	Passage history	Animal model	Notes, type of model	Reference
LASV	Josiah (Jos)	Sierra Leone (1976), human isolate	4 passages (P) in Vero cell at low MOI	Guinea pig, NHP	MCM evaluation, pathogenesis	[100]
	Soromba-R (Sor)	Mali (2010), *Mastomys natalensis *	2 P Vero E6	NHP	Pathogenesis	[101]
	Z-132	Liberia (1984), human isolate	<4 P Vero E6	NHP	Pathogenesis	[102]

JUNV	Romero (Rom) (P3235)	Argentina (1986), severe nonfatal human infection (hemorrhagic/neurological)	2 PMRC-5 cells, 1P Vero cells	Guinea pig, NHP	MCM evaluation, pathogenesis	[81]
	Romero	cDNA encoding prototypical Romero genome	Transfection BHK-21 cells, Vero cell propagation	Guinea pig	Pathogenesis	[103]
	MC2	Argentina (1967), *Calomys musculinus*,	1 P MB, 1 P Vero cells, and 1 P BHK cells	Guinea pig	Pathogenesis	[104]
	XJ	Argentina (1958), human infection	1 P mouse brain, 1 P guinea pig, 1 P mouse brain, 1 P Vero cells, and 1 P BHK cell	Guinea pig	MCM evaluation, pathogenesis	[105]
	Espindola (Esp) (P3790)	Argentina (1986), fatal human (hemorrhagic)	2 PMRC-5 cells, 1 P Vero cells	NHP	Pathogenesis, hemorrhagic disease in NHPs	[106]
	Ledesma (LED) (P3406)	Argentina (1986), fatal human (neurological)	2 PMRC-5 cells, 1 P Vero cells	NHP	Pathogenesis, neurological disease in NHPs	[106]
	Candid#1	Attenuated, vaccine strain	Derived from XJ44, passage continued 2 P guinea pig, 44 P mouse brain, and 19 P FRhL cells	Mice, Guinea pig, NHP	MCM evaluation, pathogenesis	[107]

MACV	Carvallo (Car)	Bolivia (1963), fatal human isolate	2-3 P suckling hamster brain	Guinea pig, NHP	MCM evaluation, pathogenesis	[94]
	Chicava (Chi)	Bolivia (1993), fatal human isolate	2 P Vero E6	Guinea pigs, NHP	Pathogenesis	[97]

GTOV	95551	Venezuela (1990), human isolate	2 P mouse brain, 1 passage Vero cells	Guinea pigs	MCM evaluation, pathogenesis	[98, 99]

LUJO	Wild-type	South Africa (2008), human isolate	5 P Vero E6	Guinea pigs, NHP	Pathogenesis	[108]
	Recombinant	cDNA derived from wild-type virus	Transfection BHK-21 cells, Vero cell propagation	Guinea pigs	Pathogenesis	[108]

**Table 2:** Animal models for the evaluation of arenavirus pathogenesis and countermeasure protective efficacy.

	Virus	Animal model/strain	Virus strain	Virus dose	Route(s) of infection	% lethality	Time to death (days)	Salient features	Reference
OW	LASV	Guinea pig/Strain 13	Jos, Z-132, and Sor	≥2 PFU, 1 × 10^4^ TCID_50_ some studies	s.c., i.p., and i.m.	60% (Sor)–100% (Jos, Z-132)	11–22 (Jos, Z-132), >17 days (Sor)	interstitial pneumonia, Sor strain less virulent	[101, 102, 109]
		Guinea pig/Hartley	Jos	≥2 PFU	s.c., i.p.	<30% (s.c.) ~67% (i.p.)	15–24	Viremia, seroconversion	[109, 110]
		Macaque	Jos, Z-132, and Sor	1 × 10^4^ TCID_50_	i.m., aerosol	57% (Sor)–100% (Jos, Sor)	12–19	Viremia, interstitial pneumonia, vasculitis, and occasional virus shedding	[101, 110–117]
		Marmoset	Jos	1 × 10^3^–1 × 10^6^ PFU	s.c.	Unknown (animals euthanized)	Unknown (severe morbidity 15–20 days)	Hepatic necrosis, lymphoid depletion, and interstitial nephritis	[118, 119]
		*Mastomys natalensis *	Unclear	Unclear	i.p.	None	None	Persistent infection	[110]
	LUJV	Guinea pig/Strain 13	NA	10–100,000 FFU	i.p.	100%	11–16	Weight loss, virus in multiple organs, leukopenia, thrombocytopenia, elevated transaminase levels, and animal age 52–78 weeks	[108]
		Macaques	NA	10,000 TCID_50_	i.m.	Not lethal	Not lethal	Mild illness, delayed inflammatory response	[120]

NW	JUNV	Guinea pig/Strain 13	Rom, XJ	1,000–6,000 PFU	i.p.	0–100% (strain specific)	100% (Rom), 0% (XJ)	Weight loss, elevates AST/PLT levels, thrombocytopenia	[83]
		Guinea pig/Hartley	Multiple, Rom (most lethal)	1–5,000 PFU	i.p., i.n., i.m., and oral	0–100% (strain specific)	Hemorrhagic strains; 12–19, neurological strains; >20, attenuated strains; no death	Strain dependent: neurological and/or hemorrhagic, leukopenia, thrombocytopenia, and interferon-alpha production	[79–83, 85, 89–93]
		Neonatal mice	XJ13, can#1, reassert, and gene replacements	500 TCID_50_	i.c.	0–100%	<28	Death is inconsistent with human disease, strain Can#1 is not lethal	[121]
		Mice/type I IFN *α*/*β* and gamma receptor KO	Rom	10,000 PFU	i.p.	Unclear; all euthanized with severe disease	~13.5 (unclear; all euthanized with severe disease)	Viremia, viral dissemination	[122]
		Macaque	Rom, Esp, and Led	10,000–100,000 PFU	i.m.	0% (Rom) 100% (Esp)	Day 33 (Esp)	Espindola; hemorrhagic, Ledesma; neurological manifestations	[106, 107, 123–126]
		Marmoset	XJ	1,000 TCID_50_	i.m.	100%	22–32	Anorexia, decrease in weight, leukopenia, thrombocytopenia, viremia, hemorrhage of the gums, pharynx, esophagus abdominal cavity, and neurological illness	[127–130]
		*Calomys musculinus *	An9446	100 TCID_50_	i.n.	~70% (newborn) ~100% fetus	Day 24–40 (newborn)	Persistent infection model, impacts adult fertility	[131–134]
	MACV	Guinea pig/Hartley	Car, Chi	10,000 PFU	i.p.	0–80%	18–23 days	Weight loss, decreased activity, and strain 13 animals also attempted	[94–97]
		Mice/type I IFN *α*/*β* and gamma receptor KO	Car, recombinant Car	10,000 PFU	i.p.	100%	23-24	Viremia, neuronal damage, and recombinant virus as lethal as wild-type	[135]
		Mice/STAT KO	Car	1,000 PFU	i.p.	100%	7.3	Elevated cytokines	[136]
		Macaque	Car, Chi	1,000–100,000 PFU	i.m., aerosol	100% (some euthanized with severe disease)	16–21	Hemorrhagic, facial edema, anorexia, tremor, and photophobia (one animal)	[97, 137–140]
		*Calomys callosus *	Unknown	100–10,000 PFU	i.p.	0%	NA	Persistent infection model Higher challenge doses favor clearance of virus	[96, 141]
	GTOV	Guinea pig/Hartley	95551	1,000 PFU	i.p.	100%	12–18	Weight loss, hemorrhage	[98]
		Guinea pig/Strain 13	95551	1,000 PFU	i.p.	100%	12–18	Weight loss, hemorrhage	[98, 99]
	PICV	Hamster/LVA (Syrian golden; outbred)	AN 3739, AN 4763	35–3.5 × 10^6^ PFU	i.p., s.c.	Lethality only in <8-day-old animals unless immunosuppressed	6–35 in <8-day-old hamsters 7–18 immunosuppressed adult animals	Humoral response, adults clear virus	[142, 143]
		Hamster/MHA (inbred)	AN 3739	35 PFU–3.5 × 10^6^ PFU	i.p.	>70% lethality	8–22	Peak viremia day 8, higher challenge dose led to >90% lethality, and humoral response	[142]
		Guinea pig/strain 13	AN 4763	0.003–3000 PFU	s.c.	0–100%	13–18	P18 virus lethal, P2 not lethal	[144]
		Guinea pig/Hartley	AN 4763	0.003–3000 PFU	s.c.	0%–43%	8–18	Only high doses lethal	[144]

**Table 3:** Animal models for the evaluation of hantavirus pathogenesis.

	Virus	Animal model/strain	Virus strain	Virus dose	Route(s) of infection	% lethality	Time to death (days)	Salient features	Reference
OW	HTNV	ICR suckling mice	76–118	830 PFU/mL	i.c., i.p., i.m., and s.c.	100	19-20	Only in mice infected within 72 hrs of birth, decreasing thereafter	[177]
	HTNV	ICR suckling mice	76–118	Serial passages of homogenized brain	i.c., i.p.	97	13-14	Mice infected 2 to 4 days after birth	[184, 185]
	HTNV	BALB/c suckling mice	76–118	3.8 × 10^3^ PFU (IP), 7.5 × 10^2^ PFU (IC)	i.c., i.p.	22.2 by both routes	8–15	Mice infected within 24 hrs of birth	[186]
	HTNV	Striped field mice	76–118	10^5.9^ ID_50_	i.m., s.c., i.p., p.o., i.l., and i.n.	0	N/A	Antigen, not infectious virus, and persists in lung for 1 yr	[187]
	HTNV	6–10-week-old Syrian hamsters	76–118	10^3^ PFU	i.m.	0	N/A	Asymptomatic infection	[188–190]
	PUUV	6–10-week-old Syrian hamsters	K27	1,000–2,000 PFU	i.m.	0	N/A	Asymptomatic infection	[46, 188, 190]
	PUUV	Suckling and weanling bank voles	Hällnäs	750–10^3.5^ ID_50_	i.c., i.m.	0	N/A	Asymptomatic, persistent infection	[191]
	PUUV	Cynomolgus macaques	Hällnäs	10^5^ ID_50_	i.t.	0	N/A	NHP exhibit lethargy, proteinuria, and microhematuria, with histopathological changes in the kidney	[192]
	DOBV	1–5-day-old NMRI suckling mice	Slovenia	50, 500, and 5,000 FFU	i.c.	13–88	18–26	Viremia, neutralizing antibodies, and elevated levels of NO detected	[193]
	DOBV	6–8-week-old Syrian hamsters	Slovenia	2,000 PFU	i.m.	0	N/A	Asymptomatic infection	[188]
	SEOV	6-day-old Lewis rats	80–39	10^6^ TCID_50_	i.p.	0	N/A	Asymptomatic, persistent infection	[194]
	SEOV	Syrian hamsters (no age specified)	80–39	1,000 PFU	i.m.	0	N/A	Asymptomatic infection	[190]
	SEOV	70–80-day-old Norway rats	SR-11	10^−4^–10^6^ PFU	i.p.	0	N/A	Asymptomatic, persistent infection	[195]
	SEOV	Syrian hamsters (no age specified)	SR-11	1,000 PFU	i.m.	0	N/A	Asymptomatic infection	[196]

NW	ANDV	6–8-week-old Syrian hamsters	Chile-9717869	2–20,000 PFU	i.m., i.n., s.c., and i.g.	Up to 100	10–16 IM, 10–20 IN	Recapitulates human disease in incubation time, rapid-progressing respiratory distress, and pathologic findings in the lung	[62, 197, 198]
	SNV	6–8-week-old Syrian hamsters	CC107	2,000–20,000 PFU	i.m.	0	N/A	Asymptomatic infection, no viremia, and little dissemination Hamster-adapted SNV increases dissemination	[197, 199, 200]
	SNV	6–8-week-old Syrian hamsters	CC107	2,000 PFU	i.m.	100	10–14	Hamsters immunosuppressed with dexamethasone and cyclophosphamide	[201]
	SNV	Rhesus macaques	77734	6 × 10^6^ based on RT-PCR Ct values	i.t., i.n., p.o., and intraocular simultaneously	70	15–22	Using only deer mouse-passaged SNV	[202]
	SNV	4–6-week-old deer mice	77734	5–20 ID_50_	i.m.	0	N/A	Asymptomatic, persistent infection	[203, 204]
	MAP	4-week-old Syrian hamsters	97021050	3.1 log_10_⁡ CCID_50_	i.m.	30	9–13	Recapitulates human disease in incubation time, rapid-progressing respiratory distress, and pathologic findings in the lung	[205]
	PHV	Cynomolgus macaques/Chimpanzee	Prospect Hill I	10^7^ PFU	i.v.	0	N/A	Mild, transient proteinuria	[206]

**Table 4:** Prototypical hantavirus strains and their origin.

Virus	Strain	Origin	Passage history	Animal model	Notes, type of model	Reference
HTNV	76–118	Korea (1978), *Apodemus agrarius *	8 P *Apodemus agrariu*s	*Apodemus agrariu*s	Persistent infection model	[187]
			5 P *Apodemus agrariu*s, 3-4 P A549 cells, 2-18 P Vero E6	ICR suckling mice	MCM, pathogenesis	[177, 184–186, 188–190]

PUUV	Hällnäs	Sweden (1981), *Clethrionomys glareolus *	2 P *Clethrionomys glareolus *	*Clethrionomys glareolus*, NHP	Persistent infection model, pathogenesis	[191, 192, 207]
	K27	U.S.S.R (1983), *Clethrionomys glareolus *	5 P Vero E6	Syrian hamster	MCM, pathogenesis	[208]

DOBV	Slovenia	Yugoslavia (1992), *Apodemus flavicollis *	5 P Vero E6	NMRI suckling mice, Syrian hamster	MCM, pathogenesis	[209]

SEOV	80–39		3 P Wistar rats, 8 P Vero E6	Syrian hamster, Lewis rat, and Norway rat	MCM, persistent infection model	[210]
	SR-11	Japan (1983), Rat	5–7 P Vero E6	Syrian hamster, Norway rat	MCM, persistent infection model	[211]

ANDV	Chile-9717869	Chile (1997), *Oligoryzomys longicaudatus *	4–7 P Vero E6	Syrian hamster	MCM, pathogenesis	[197, 212]

SNV	CC107	United States (1995), *Peromyscus maniculatus *	3-4 P Vero E6	Syrian hamster	MCM, pathogenesis	[213]
	77734	United States (2000), *Peromyscus maniculatus *	2 P *Peromyscus maniculatus *	*Peromyscus maniculatus*, NHP	Pathogenesis	[203]

MAP	97021050	Venezuela (1997), *Oecomys bicolor *	4 P Vero E6	Syrian hamster	Pathogenesis	[205]

PHV	Prospect Hill I	United States (1982), *Microtus pennsylvanicus *	1 P *Microtus pennsylvanicus*, Serial P Vero E6	NHP	Pathogenesis	[214]
