# Supplementary material for: Animal Models for the Study of Rodent-Borne Hemorrhagic Fever Viruses: Arenaviruses and Hantaviruses
Source: Biomed Res Int. 2015 Jul 21;2015:793257. doi: 10.1155/2015/793257 (PMC4523679; doi:10.1155/2015/793257)
Supplement: Supplementary file 1 — Hamsters represent an important small animal model to study a variety of fields including metabolism, carcinogenesis/tumorigenesis, inflammation and infection and disease. However, hamster models are often overlooked by many investigators, resulting in a lower popularity when compared to mouse and rat models. In some cases, as with New World hantaviruses, hamsters represent the only known small animal model of human disease caused by these viruses, elevating their importance as a tool to understand human disease pathogenesis. Still, the dearth of hamster-specific reagents to study many aspects of disease pathogenesis and immunological processes, when compared to the overwhelming abundance of mouse, rat, human, and nonhuman primate reagents, has made a direct comparison of human disease and hamster disease, difficult. In rare instances, commercial vendors such as MyBioSource.com have begun selling hamster-specific reagents. Unfortunately, this is more the exception than the rule as most reagents used to study hamsters are cross-reactive mouse, rat and human reagents that have been identified by trial and error. In the following table, we have attempted to consolidate those, predominantly immunologically related, reagents that we and others have used in the hamster model. [file 793257.f1.pdf]

Supplemental information

Immunological reagents for non-murine rodents. The use of Syrian hamsters to model biological diseases is not unique to hantaviruses. Hamsters have been considered a valuable model to study the disease caused by many human pathogens including Toxocara canis (1), Nipah virus (2, 3), measles virus (4), prion diseases (5), Leishmania (6) Yellow fever virus (7), SARS (8, 9), West Nile virus (10, 11), encephalitis viruses (12-15) and Ebola virus (16), due to the numerous similarities between the disease manisfested in hamsters and the natural course of human disease pathogenesis. However, the hamster model is unique from its mouse and rat counterparts in that, despite pioneering studies in the 1980s identifying lymphocyte subsets in the Syrian hamster (17-21) and the cloning of multiple hamster cytokine genes in 2000 (22), there have been relatively few advances in the development and characterization of commercially available reagents to study immunological mechanisms in the hamster. The identification of anti-mouse CD4 (17, 23) and anti-rat CD8 antibodies that cross-react with orthologous proteins expressed on hamster CD4 and CD8 T cells have made it possible to global T cell responses to ANDV infection in hamsters by flow cytometry)(24, 25). In these studies, T cell activation and accumulation in the lung correlated with the onset of disease in hamsters but the depletion of CD4 and/or CD8 T cell subsets in hamsters did not prevent disease. However, the majority of mouse and rat specific for other T cell specific surface molecules (i.e. activation markers, adhesion markers, inhibitory ligands, memory markers) or intracellular cytokines do not cross react with their hamster ortholog, or have not been identified, making detailed analysis of T cell responses in hamsters difficult. More recently, an antibody specific for the hamster CD3 antigen has been developed and used to deplete hamster T cells in a model of tumor immunity (26) but this antibody is not commercially available. With the exception of one antibody that identifies

the MARCO scavenger receptor on Syrian hamster alveolar macrophages(27), flow cytometry antibodies specific for most hamster immune cell types (e.g. neutrophils, monocytes, NK cells, dendritic cells) are scarce. A handful of hamster-specific monoclonal antibodies, or cross-reactive monoclonal antibodies, to several common lymphocyte antigens (CD18, CD4, CD8, MHC I) as well as three antibodies that more broadly identify hamster immune cells (T cell, B cell and lymphocyte) are available from Kingfisher Biotech, Inc. Along with flow cytometry assays, the use of mouse and rat assays that rely on conformational protein epitopes, such as ELISA and Luminex assays, have been problematic in their ability to accurately recognize hamster proteins. While a few commercially available mouse and rat ELISA and Luminex assays have been found to suitably cross-react with hamster samples, many produce uncharacteristically abnormal measurements or fail to cross-react altogether (28). Notwithstanding, within the last several years a variety of hamster-specific cytokine ELISA assays have recently become available by the online company MyBioSource. Results using these kits have yet to be reported in the literature but we have found that hamster protein concentrations detected by kits from various tissue sources (e.g. serum, lung homogenates, BAL fluid) to fall within the acceptable limits of the standards. Antibodies that cross-react with linear hamster protein eptitopes have been more successful in identifying the expression of numerous proteins by western blot analysis such as caspases, matrixmetalloproteinases, autophagy pathway member proteins such as ubiquitin and proteins involved in inflammatory cytokine responses such as NF-κB, JAK2 and STAT3 (29-35). Similarly, expanded sets of PCR primers for over 70 hamster proteins have been published (22, 28, 36) and used in hamster models of disease pathogenesis and oncology and the recent sequencing, annotation and analysis of the Syrian hamster transcriptome(37) should facilitate the expansion of a new generation of resources to

study the hamster model. A list of reagents compatible with the Syrian hamster, including cross reactive flow cytometry and western blot antibodies, cross-reactive ELISA and Luminex assays, cytokines and PCR primers, is shown in **Table S1**.

Table S1. Immunological reagents specific for, or known to cross-react with, the Syrian hamster.

**Western Blot Antibodies**			
Antigen	***Tissue(s) Analyzed***	***Antigen***	***Tissue(s) Analyzed***
**Akt** (phospho)	Heart(1)	**MMP-9**	Buccal pouch(2, 3)
**Apaf-1**	Buccal pouch(2)	**NFκB-p50**	Buccal pouch(2)
**Bax**	Buccal pouch(2, 3)	**NF-κB-p65**	Buccal pouch(2)
**Bcl-2**	Buccal pouch(2, 3)<br>Liver(4)	**p21^{waf-1}**	Buccal pouch(2, 3)
**Bcl-xL**	Liver(4)	**p53**	Buccal pouch(2, 3)
**c-fos**	Liver(4)	**p65**	Liver(4)
**c-jun**	Liver(4)	**p65** (phospho)	Liver(4)
**Caspase-2L**	Buccal pouch(2, 3)	**PARP**	Buccal pouch(3)<br>Liver(4)
**Caspase-3**	Buccal pouch(2, 3)<br>Liver(4)<br>Endometrium(5)	**PARP** (cleaved)	Liver(4)
**Caspase-6**	Buccal pouch(2, 3)	**Procaspase 3**	Liver(4)
**Caspase-8**	Buccal pouch(2, 3)<br>Liver(4)	**Procaspase 8**	Liver(4)
**Caspase-9**	Buccal pouch(2, 3)<br>Liver(4)	**Procaspase 9**	Liver(4)
**Cathepsin D**	Heart(1)	**Rab7**	Heart(1)
**COX-2**	Buccal pouch(6)<br>Liver(4)	**RECK**	Buccal pouch(3)
**Cytochrome C**	Buccal pouch(2, 3)	**STAT-3**	Liver(4)
**ERK** (phospho)	Heart(1)	**STAT-3** (phospho)	Liver(4)<br>Heart(1)
**Fas**	Buccal pouch(2, 3)	**Survivin** (C)	Buccal pouch(3)
**HIF-1**	Buccal pouch(2, 3)	**Survinin** (N)	Buccal pouch(3)
**ICAM-1**	Lung(7)	**TIMP-2**	Buccal pouch(3)
**IκB**	Buccal pouch(2)	**TNFα**	Heart(1)
**iNOS**	Liver(4)	**TRAF1**	Liver(4)
**JAK2**	Liver(4)	**Ubiquitin**	Heart(1)
**Mcl-1**	Buccal pouch(3)	**VCAM-1**	Lung(7)
**MMP**	Heart(1)	**VE-cadherin**	Lung(7)
**MMP-2**	Buccal pouch(2) (3)	**VEGF**	Buccal pouch(2) (3)<br>Endometrium(5)

**Immunohistochemistry Antibodies**			
Antigen	***Tissue(s) Analyzed***	***Antigen***	***Tissue(s) Analyzed***
**Akt** (phospho)	Heart(1)	**iNOS**	Liver(4)
**Bax**	Buccal pouch(2, 3, 8)	**MMP-2**	Uterus(9)
**Bcl-2**	Buccal pouch(2, 3, 8)	**p53**	Buccal pouch(8)
**Caspase-3**	Buccal pouch(2)	**p65**	Liver(4)
**Cathepsin D**	Heart(1)	**MARCO**	Lung(10)
**COX-2**	Buccal pouch(6)	**RECK**	Buccal pouch(2)
**factorVIII/von**	Buccal pouch(6)	**Stat3** (phospho)	Heart(1)

**Willebrand factor**	Heart(1)		
**Flk-1**	Vascular endothelial cells(11)<br>Uterus(9)	**TUNEL**	Heart(1)
**FLT-1**	Uterus(9)	**VEGF**	Buccal pouch(8)<br>Vascular endothelial cells(11)<br>Uterus(9)

**Flow Cytometry / In Vivo Depletion Antibodies**			
Antigen	***Tissue(s) Analyzed***	***Antigen***	***Tissue(s) Analyzed***
**Annexin V** (mammalian)	Spleen/PBMC/lung(12)	**FoxP3** (FJK-16s; mouse/rat)	Spleen/lung (Hammerbeck unpublished)
**BrdU** (3D4; human)	Spleen/PBMC/lung(12)	**MHC II** (14-4-4S; mouse I-E^k/rat RT1D)	Spleen/PBMC/lung(12)<br>Spleen(13) (14)
**CD3** (4F11; mouse)	Lymphoid/nonlymphoid tissue(15)	**MARCO** (PAL-1; hamster)	Lung/BAL(10)
**CD4** (GK1.5; mouse)	Spleen/PBMC/lung(12)<br>Spleen(16) (14)	**TNFα** (MP6-XT22; mouse)	Spleen (Hammerbeck unpublished)
**CD8β** (341; rat)	Spleen/PBMC/lung(12)<br>Spleen(14)		

**Recombinant Cytokines**			
Cytokine	***Reference***	***Cytokine***	***Reference***
**G-CSF** (human)	Miyata(1)	**Platelet-activating factor (PAF)**	Kim(17)
**GM-CSF** (human)	Parviainen(18)	**TNFα** (mouse)	Falcinelli et.al.(7)
**IL-2** (mouse)	Sobotkova(19)	**$VEGF_{165}$** (human)	Kim(17)<br>Aramoto(20)
**IL-12** (mouse)	Bortolanza(21)		

**ELISA Kits**			
Analyte	***Tissue(s) Analyzed***	***Analyte***	***Tissue(s) Analyzed***
**c-fos**	Buccal pouch(8)	**PGE2**	Buccal pouch(8)
**COX-2**	Buccal pouch(8)	**TGF-β** (rat; marginal)	Plasma(22)
**Caspase-3, -9**	Buccal pouch(8)	**IL-10** (rat; marginal)	Plasma(22)
**VEGF** (human)	Buccal pouch(8)	**IL-1β** (mouse; marginal)	Plasma(22)

**Lumiex Panels**			
Analyte	***Tissue(s) Analyzed***	***Analyte***	***Tissue(s) Analyzed***
**GM-CSF** (rat)	Plasma(22)	**MIG**	Plasma(22)
**Leptin** (rat)	Plasma(22)	**IL-13**	Plasma(22)
**GRO/KC** (rat)	Plasma(22)	**Eotaxin** (mouse; marginal)	Plasma(22)
**IL-1α** (rat)	Plasma(22)	**MIP-1β** (mouse; marginal)	Plasma(22)
**RANTES** (rat; marginal)	Plasma(22)	**IL-12p40** (mouse; marginal)	Plasma(22)
**IL-10** (rat; marginal)	Plasma(22)		

**PCR Primers**			
Protein	***Tissue(s) Analyzed***	***Protein***	***Tissue(s) Analyzed***
**Apaf-1**	Buccal pouch(3)	**IL-21**	Spleen, liver, lung(22)
**β2 microglobulin**	Spleen, liver, lung(22)	**IL-2Rα**	Spleen, liver, lung(22)
**β-actin**	Spleen, liver, lung(22)	**IL-6 signal transducer**	Spleen, liver, lung
**Bad**	Buccal pouch(3)	**iNOS**	Spleen, liver, lung(22)<br>Spleen(23)

**Bax**	Buccal pouch(2) (3)	**IP-10**	Spleen, liver, lung(22)
**Bcl-2**	Buccal pouch(2) (3)<br>Spleen, liver, lung(22)	**IRF1**	Spleen, liver, lung(22)
**Bcl-2 associated protein**	Spleen, liver, lung(22)	**IRF2**	Spleen, liver, lung(22)
**Bcl-xL**	Buccal pouch(3)	**Junction adhesion molecule**	Spleen, liver, lung(22)
**Bid**	Buccal pouch(3)	**MMP-2**	Spleen, liver, lung(22)<br>Buccal pouch(2) (3)
**CCL20/MIP-3a**	Spleen, liver, lung(22)	**MMP-9**	Buccal pouch(2) (3)
**CCL17**	Spleen, liver, lung(22)	**MHC II alpha chain**	Spleen, liver, lung(22)
**CCL22**	Spleen, liver, lung(22)	**Muc1**	Spleen, liver, lung(22)
**CD83**	Spleen, liver, lung(22)	**Myxovirus resistance protein-2**	Spleen, liver, lung(22)
**c-FLIP**	Buccal pouch(3)	**NOS2**	Spleen, liver, lung(22)
**Claudulin-1**	Spleen, liver, lung(22)	**Occuldin**	Spleen, liver, lung(22)
**Complement C3**	Spleen, liver, lung(22)	**p21**	Buccal pouch(3)
**Complement C5**	Spleen, liver, lung(22)	**P75 TNF membrane receptor**	Spleen, liver, lung(22)
**Complement protein C1q**	Spleen, liver, lung(22)	**PCNA**	Buccal pouch(3)
**Cyclin D1**	Buccal pouch(3)	**PECAM**	Spleen, liver, lung(22)
**Cyt-C**	Buccal pouch(3)	**PIGF**	Buccal pouch(3)
**E-cadherin**	Spleen, liver, lung(22)	**Protein kinase R**	Spleen, liver, lung(22)
**Fas-L**	Buccal pouch(3)	**Ribosomal Protein L18**	Spleen, liver, lung(22)
**Fibrinogen**	Spleen, liver, lung(22)	**STAT 1**	Spleen, liver, lung(22)
**FoxP3**	Spleen, liver, lung(22)	**STAT1b**	Spleen, liver, lung(22)
**GST-P**	Buccal pouch(3)	**STAT2**	Spleen, liver, lung(22)
**Hypoxanthine phosphoribosyltransferase**	Spleen, liver, lung(22)	**Tight junction protein 2**	Spleen, liver, lung(22)
**ICAM-1**	Spleen, liver, lung(22)	**TIMP-2**	Spleen, liver, lung(22)<br>Buccal pouch(3)
**IFNα inducible protein**	Spleen, liver, lung(22)	**TGF-β**	Spleen(24) (23)
**IFNγ**	Spleen, liver, lung(22)<br>Spleen(24) (23)	**TGF-β1**	Spleen, liver, lung(22)
**IL-1β**	Spleen, liver, lung(22)	**TGF-β2**	Spleen, liver, lung(22)
**IL-2**	Spleen, liver, lung(22)<br>Spleen(24) (23)	**TGF-β3**	Spleen, liver, lung(22)
**IL-4**	Spleen, liver, lung(22)<br>Spleen(24) (23)	**TGF-β1R**	Spleen, liver, lung(22)
**IL-6**	Spleen, liver, lung(22)	**TNFα**	Spleen, liver, lung(22)<br>Spleen(24) (23)
**IL-10**	Spleen, liver, lung(22)<br>Spleen(24) (23)	**VEGF**	Spleen, liver, lung(22)<br>Buccal pouch(2) (3)
**IL-12 p35**	Spleen, liver, lung(22)	**VEGFR1**	Buccal pouch(3)
**IL-20p40**	Spleen, liver, lung(22)<br>Spleen	**VEGFR2**	Buccal pouch(3)

References

1. **Miyata S, Takemura G, Kawase Y, Li Y, Okada H, Maruyama R, Ushikoshi H, Esaki M, Kanamori H, Li L, Misao Y, Tezuka A, Toyo-Oka T, Minatoguchi S, Fujiwara T, Fujiwara H.** 2006. Autophagic cardiomyocyte death in cardiomyopathic hamsters and its prevention by granulocyte colony-stimulating factor. The American journal of pathology **168:**386-397.

2. **Nagini S, Letchoumy PV, A T, Cr R.** 2009. Of humans and hamsters: a comparative evaluation of carcinogen activation, DNA damage, cell proliferation, apoptosis, invasion, and angiogenesis in oral cancer patients and hamster buccal pouch carcinomas. Oral oncology **45:**e31-37.
3. **Priyadarsini RV, Vinothini G, Murugan RS, Manikandan P, Nagini S.** 2011. The flavonoid quercetin modulates the hallmark capabilities of hamster buccal pouch tumors. Nutrition and cancer **63:**218-226.
4. **Prakobwong S, Khoontawad J, Yongvanit P, Pairojkul C, Hiraku Y, Sithithaworn P, Pinlaor P, Aggarwal BB, Pinlaor S.** 2011. Curcumin decreases cholangiocarcinogenesis in hamsters by suppressing inflammation-mediated molecular events related to multistep carcinogenesis. International journal of cancer. Journal international du cancer **129:**88-100.
5. **Laschke MW, Elitzsch A, Scheuer C, Holstein JH, Vollmar B, Menger MD.** 2006. Rapamycin induces regression of endometriotic lesions by inhibiting neovascularization and cell proliferation. British journal of pharmacology **149:**137-144.
6. **Segawa E, Hashitani S, Toyohara Y, Kishimoto H, Noguchi K, Takaoka K, Urade M.** 2009. Inhibitory effect of sulindac on DMBA-induced hamster cheek pouch carcinogenesis and its derived cell line. Oncology reports **21:**869-874.
7. **Falcinelli S, Gowen BB, Trost B, Napper S, Kusalik A, Johnson RF, Safronetz D, Prescott J, Wahl-Jensen V, Jahrling PB, Kindrachuk J.** 2015. Characterization of the Host Response to Pichinde Virus Infection in the Syrian Golden Hamster by Species-Specific Kinome Analysis. Molecular & cellular proteomics : MCP.
8. **Silvan S, Manoharan S.** 2013. Apigenin prevents deregulation in the expression pattern of cell-proliferative, apoptotic, inflammatory and angiogenic markers during 7,12-dimethylbenz[a]anthracene-induced hamster buccal pouch carcinogenesis. Archives of oral biology **58:**94-101.
9. **Chow PH, Jiang HY, Poon HK, Lee KH, O WS.** 2003. Embryos sired by males without accessory sex glands induce failure of uterine support: a study of VEGF, MMP and TGF expression in the golden hamster. Anatomy and embryology **206:**203-213.
10. **Palecanda A, Paulauskis J, Al-Mutairi E, Imrich A, Qin G, Suzuki H, Kodama T, Tryggvason K, Koziel H, Kobzik L.** 1999. Role of the scavenger receptor MARCO in alveolar macrophage binding of unopsonized environmental particles. The Journal of experimental medicine **189:**1497-1506.
11. **Yi XJ, Jiang HY, Lee KK, O WS, Tang PL, Chow PH.** 1999. Expression of vascular endothelial growth factor (VEGF) and its receptors during embryonic implantation in the golden hamster (Mesocricetus auratus). Cell and tissue research **296:**339-349.
12. **Hammerbeck CD, Hooper JW.** 2011. T cells are not required for pathogenesis in the Syrian hamster model of hantavirus pulmonary syndrome. Journal of virology **85:**9929-9944.
13. **Liu H, Steiner BM, Alder JD, Baertschy DK, Schell RF.** 1990. Immune T cells sorted by flow cytometry confer protection against infection with Treponema pallidum subsp. pertenue in hamsters. Infection and immunity **58:**1685-1690.
14. **Prescott J, Safronetz D, Haddock E, Robertson S, Scott D, Feldmann H.** 2013. The adaptive immune response does not influence hantavirus disease or persistence in the Syrian hamster. Immunology **140:**168-178.
15. **Tysome JR, Li X, Wang S, Wang P, Gao D, Du P, Chen D, Gangeswaran R, Chard LS, Yuan M, Alusi G, Lemoine NR, Wang Y.** 2012. A novel therapeutic regimen to eradicate established solid tumors with an effective induction of tumor-specific immunity. Clinical cancer research : an official journal of the American Association for Cancer Research **18:**6679-6689.
16. **Dondji B, Bungiro RD, Harrison LM, Vermeire JJ, Bifulco C, McMahon-Pratt D, Cappello M.** 2008. Role for nitric oxide in hookworm-associated immune suppression. Infection and immunity **76:**2560-2567.

17. **Kim DD, Kleinman DM, Kanetaka T, Gerritsen ME, Nivaggioli T, Weber D, Duran WN.** 2010. Rapamycin inhibits VEGF-induced microvascular hyperpermeability in vivo. Microcirculation **17:**128-136.
18. **Parviainen S, Ahonen M, Diaconu I, Kipar A, Siurala M, Vaha-Koskela M, Kanerva A, Cerullo V, Hemminki A.** 2015. GMCSF-armed vaccinia virus induces an antitumor immune response. International journal of cancer. Journal international du cancer **136:**1065-1072.
19. **Sobotkova E, Indrova M, Simova J, Bubenik J, Vonka V.** 1996. Cross-reactivity of murine and hamster IL-2 mitogenic signal. Immunology letters **50:**115-117.
20. **Aramoto H, Breslin JW, Pappas PJ, Hobson RW, 2nd, Duran WN.** 2004. Vascular endothelial growth factor stimulates differential signaling pathways in in vivo microcirculation. American journal of physiology. Heart and circulatory physiology **287:**H1590-1598.
21. **Bortolanza S, Bunuales M, Otano I, Gonzalez-Aseguinolaza G, Ortiz-de-Solorzano C, Perez D, Prieto J, Hernandez-Alcoceba R.** 2009. Treatment of pancreatic cancer with an oncolytic adenovirus expressing interleukin-12 in Syrian hamsters. Molecular therapy : the journal of the American Society of Gene Therapy **17:**614-622.
22. **Zivcec M, Safronetz D, Haddock E, Feldmann H, Ebihara H.** Validation of assays to monitor immune responses in the Syrian Golden hamster (Mesocricetus auratus). J Immunol Methods.
23. **Gupta R, Kushawaha PK, Samant M, Jaiswal AK, Baharia RK, Dube A.** 2012. Treatment of Leishmania donovani-infected hamsters with miltefosine: analysis of cytokine mRNA expression by real-time PCR, lymphoproliferation, nitrite production and antibody responses. The Journal of antimicrobial chemotherapy **67:**440-443.
24. **Melby PC, Tryon VV, Chandrasekar B, Freeman GL.** 1998. Cloning of Syrian hamster (Mesocricetus auratus) cytokine cDNAs and analysis of cytokine mRNA expression in experimental visceral leishmaniasis. Infection and immunity **66:**2135-2142.
